# Analysis of *IDH1* and *IDH2* mutations as causes of the hypermethylator phenotype in colorectal cancer

**DOI:** 10.1002/path.6446

**Published:** 2025-06-22

**Authors:** Joseph C Ward, Melissa Morgan, James Wood, Connor Woolley, Albert Antao Nobre de Menezes, Alina Finch, Kitty Sherwood, Qiwen Huang, Chloe S Henry, Juan Fernández‐Tajes, Ignacio Soriano, Steve Thorn, Isabelle Legge, James McCullagh, David Kerr, Rachel Kerr, Rahul K Hejmadi, Mark J Arends, The S:CORT Consortium, Richard Adams, Simon Bach, Andrew Beggs, Louise Brown, Francesca Buffa, Jean‐Baptiste Cazier, Enric Domingo, Andrew Blake, Che‐Hsi Wu, Ekaterina Chatzpili, Susan Richman, Philip Dunne, Paul Harkin, Geoff Higgins, Jim Hill, Chris Holmes, Denis Horgan, Rick Kaplan, Richard Kennedy, Mark Lawler, Simon Leedham, Ultan McDermott, Gillies McKenna, Gary Middleton, Dion Morton, Graeme Murray, Philip Quirke, Manuel Salto‐Tellez, Les Samuel, Anna Schuh, David Sebag‐Montefiore, Matt Seymour, Ricky Sharma, Richard Sullivan, Ian Tomlinson, Nicholas West, Richard Wilson, Enric Domingo, Timothy Maughan, Chiara Bardella, Ian Tomlinson

**Affiliations:** ^1^ Department of Oncology University of Oxford Oxford UK; ^2^ Edinburgh Cancer Research, Cancer Research UK Scotland Centre, Institute of Genetics & Cancer University of Edinburgh, Western General Hospital Edinburgh UK; ^3^ Institute of Cancer & Genomic Sciences University of Birmingham Birmingham UK; ^4^ Department of Chemistry University of Oxford Oxford UK; ^5^ Nuffield Department of Clinical Laboratory Sciences University of Oxford, John Radcliffe Hospital Oxford UK; ^6^ Queen Elizabeth Hospital University Hospitals Birmingham NHS Foundation Trust Birmingham UK

**Keywords:** colorectal cancer, epigenetics, DNA methylation, cancer driver genes, isocitrate dehydrogenase, cancer genomics

## Abstract

The CpG island methylator phenotype (CIMP) occurs in many colorectal cancers (CRCs). CIMP is closely associated with global hypermethylation and tends to occur in proximal tumours with microsatellite instability (MSI), but its origins have been obscure. A few CRCs carry oncogenic (gain‐of‐function) mutations in isocitrate dehydrogenase *IDH1*. Whilst *IDH1* is an established CRC driver gene, the low frequency of *IDH1*‐mutant CRCs (about 0.5%) has meant that the effects and molecular covariates of those mutations have not been established. We first showed computationally that *IDH2* is also a CRC driver. Using multiple public and in‐house CRC datasets, we then identified IDH mutations at the hotspots (*IDH1* codons 132 and *IDH2* codons 140 and 172) frequently mutated in other tumour types. Somatic IDH mutations were associated with *BRAF* mutations and expression of mucinous/goblet cell markers, but not with *KRAS* mutations or MSI. All IDH‐mutant CRCs were CIMP‐positive, mostly at a high level. Cell and mouse models showed that IDH mutation was plausibly causal for DNA hypermethylation. Whilst the aetiology of hypermethylation generally remains unexplained, IDH‐mutant tumours did not form a discrete methylation subcluster, suggesting that different underlying mechanisms can converge on similar final methylation phenotypes. Although further analysis is required, IDH mutations may be the first cause of hypermethylation to be identified in a common cancer type, providing evidence that CIMP and DNA methylation represent more than aging‐related epiphenomena. Cautious exploration of mutant IDH inhibitors and DNA demethylating agents is suggested in managing IDH‐mutant CRCs. © 2025 The Author(s). *The Journal of Pathology* published by John Wiley & Sons Ltd on behalf of The Pathological Society of Great Britain and Ireland.

## Introduction

One of the most intriguing findings from large cancer sequencing projects has been the discovery of driver mutations that are frequent in some cancer types and much rarer in others [1]. Whilst some of the latter mutations may be passengers, there is good reason to suspect that many contribute towards tumorigenesis. For example, mutations in the tumour suppressor gene *APC* are very common in colorectal cancer (CRC), but rare in endometrial cancer. Nevertheless, *APC* mutations in endometrial cancer are bi‐allelic, specifically occur in cancers with polymerase proofreading deficiency, and almost never co‐occur with the more frequent *CTNNB1* mutations, which also drive Wnt activation [2]. In that specific case, it is possible that the incidence of *APC* mutations is increased over *CTNNB1* by the DNA repair defects underpinning ultra‐mutator tumours. For other driver genes that exhibit similar phenomena, however, no clear explanation is available.

Isocitrate dehydrogenase (IDH) mutations are frequent drivers in several cancer types, including glioma, acute myeloid leukaemia (AML), cholangiocarcinoma, and chondrosarcoma [3]. Changes to specific residues (*IDH1* codon 132, *IDH2* codons 140 and 172) result in neomorphic enzyme activity that produces D‐2‐hydroxyglutarate (D2HG), rather than converting isocitrate to α‐ketoglutarate (αKG) [4]. D2HG is proposed to inhibit αKG‐dependent enzymes, such as the ten‐eleven translocation (TET) family of DNA demethylases, thus promoting tumorigenesis [5]. The same IDH mutations are occasionally found in other cancers, including ~2% of melanomas [6], ~3% of prostate cancers [7], and ~1% of CRCs [8, 9]. Whitehall *et al* [10] screened for IDH mutations in a series of 166 CRCs, of which 86 had the CpG island methylator phenotype (CIMP‐positive). They found four *IDH1* ^*R132C*^ mutant tumours, all of which were CIMP‐positive and had co‐occurring *BRAF* mutations, although neither association was significant (*p =* 0.12 and *p =* 0.28, respectively, Fisher's exact test). Huang *et al* [9] studied 1,623 CRCs and found 15 IDH mutations, the presence of which was associated with old age, mucinous or signet ring morphology, and high grade. However, no DNA methylation analysis was conducted on these cancers. An association between pathogenic IDH mutations and DNA hypermethylation or CIMP in CRC has yet to be demonstrated.

In this study we investigated the clinicopathological associations, molecular correlates, and functional consequences of IDH driver mutations in CRC, using multiple genomic datasets, comprising over 6,500 CRCs from in‐house and public repositories. We subsequently created IDH‐mutant cell lines and mouse models. Overall, our results suggest that IDH mutations may cause CIMP in CRC and show that, despite their rarity, IDH mutations have potential clinical importance in CRC patients.

## Methods

### Cancer DNA sequencing datasets

Genome, exome, or driver gene panel sequencing data from primary CRCs were interrogated for somatic *IDH1*/*2* mutations. In‐house datasets comprised 666 patients from the S:CORT study [11], which undertook custom gene panel sequencing of primary CRCs, and 511 cases from QUASAR2 [12], which had been analysed using the Ion Torrent Comprehensive Cancer Panel (ThermoFisher Scientific, Waltham, MA, USA). We also extracted *IDH1*/*2* mutation data from whole‐genome sequences from 2,779 CRCs of the UK 100,000 Genomes Project (100kGP) [13]. A further 372 CRCs were available from the Hartwig Medical Foundation [14]. Sequence read mapping, variant calling, and quality control had been applied to each dataset using standard methods applicable to the technologies used. Publicly‐available exome or genome sequencing results were obtained from 623 CRCs (COADREAD) within The Cancer Genome Atlas (TCGA) [15], 619 CRCs within the Dana‐Farber (DFCI) project [16], and 1,099 primary or metastatic CRCs from the MSKCC project [17]. In each of these studies, clinical information and mutation profiles for each patient were collated from cBioPortal [18, 19].

### Driver analysis

*IDH2* was assessed for driver status in CRC using the IntOGen driver identification pipeline [20] in 3,327 TCGA‐COADREAD and 100kGP CRCs, restricting analysis to the coding genome to allow integration of both whole‐genome and whole‐exome sequencing data. Methods successfully producing output and used for driver identification as a part of the IntOGen pipeline [21, 22, 23, 24, 25, 26, 27] included dNdScv [21], OncodriveFML [22], MutPanning [25], and smRegions [27]. *p* values from the individual programs were combined using Stouffer's weighted z‐score combination method [28].

### Mutation signature extraction

Mutation signatures were extracted from whole‐genome sequencing of IDH‐mutant CRCs with SigProfilerExtractor, using existing reference signatures from the Catalogue of Somatic Mutations in Cancer (COSMIC) database (V3.2) [29].

### Methylation analysis

CpG dinucleotide methylation data for CRCs were downloaded from the TCGA GDC portal using the R package ‘*TCGAbiolinks*’ (V2.30.4) [30]. The methylation measure (*β*) for a CpG site was the ratio of the methylated and total probe intensities, ranging from 0 (low) to 1 (high). DNA methylation data for TCGA‐COADREAD CRCs included both Illumina 27K and 450K BeadChip arrays (Illumina Inc., San Diego, CA, USA). Integration of the two TCGA‐COADREAD methylation datasets was conducted using ChAMP (V2.21.1) [31] after restricting analysis to a common set of 20,618 probes. S:CORT CRCs used the Illumina 850K BeadChip and were analysed separately. For methylation clustering, we used a recursive‐partitioning algorithm to determine CIMP status for TCGA‐COADREAD CRCs using the top 10% most variable autosomal probes (*n* = 2,062) [32, 33]. Clustering on the S:CORT dataset was carried out using probes also used in TCGA‐COADREAD clustering (*n* = 1,475). In each case, this identified four major clusters, which we denoted as CIMP‐high (cluster 1), CIMP‐low (cluster 2) – collectively classed as CIMP‐positive – and CIMP‐negative (clusters 3 and 4). For all other datasets, detailed DNA methylation data were not available and, where available, the CIMP statuses reported by the individual studies were used. Significantly differentially methylated probes (DMPs) were identified using the R package *‘limma’* (V3.58.1) [34]. A Benjamini–Hochberg [35] corrected *p* value (*P*_FDR_) threshold of 0.05 was set to define significantly altered probes.

### Gene expression analysis

TCGA‐COADREAD HTseq harmonized counts from the TCGA GDC portal (‘*TCGAbiolinks*’) underwent exploratory data analysis using *‘DESeq2’* (V1.42.1) [36], revealing large variation in gene expression between sequencers, as shown previously [33]. In order to reduce variability, for this specific analysis we chose to proceed with CRCs from the TCGA‐COAD dataset, excluding rectal adenocarcinomas (READ), on the basis that all IDH‐mutant CRCs were from the TCGA‐COAD dataset. Consensus molecular subtype (CMS) calls, obtained by the Colorectal Cancer Subtyping Consortium through a combined network‐based approach and random forest classifier [33], were obtained from Synapse (www.synapse.org; ID syn2623706). Batch effect correction was conducted on vst‐transformed counts using the R package ‘*limma’* [34]. Following methods outlined by Guinney *et al* [33], outliers were removed based on sample‐to‐sample distances calculated via multidimensional scaling in ‘*limma*’ (absolute leading log_2_(Fold‐Change) > 2.5) and mean absolute difference in the R package ‘*arrayQualityMetrics*’ (V3.58.0) [37]. Differential expression analysis was performed on TMM‐normalized counts using ‘*edgeR’* (V4.0.16) [38] with batch effects incorporated into the linear model. Genes with an absolute log_2_(fold‐change) > 1 or < −1 and a *p*_FDR_ < 0.05 were classed as significantly altered. Gene‐set enrichment analysis (GSEA) was performed with signal‐to‐noise preranked data using Broad Institute GSEA v4.2.1 software and the MSigDB Hallmark and C8 gene‐set collections [39]. A custom gene‐set based on goblet cell markers from previous single‐cell RNA‐seq studies of intestinal tissues [40, 41, 42, 43] *AGR2*, *ANXA13*, *ATOH1*, *BEST2*, *C12orf57*, *CLCA1*, *CREB3L1*, *ERGIC1*, *FCGBP*, *FFAR4*, *FOSB*, *FOXA3*, *FRZB*, *GMDS*, *GSN*, *HEPACAM2*, *HPCAL1*, *ITLN1*, *KIAA1324*, *KLF4*, *KLK1*, *KRT18*, *LINC00261*, *LRRC26*, *MLPH*, *RAB27A*, *RAP1GAP*, *RASD1*, *REG4*, *REP15*, *RNASE1*, *RPL10*, *RPL36*, *RPL38*, *RPS11*, *RPS12*, *RPS29*, *SERF2*, *SERPINA1*, *SH3BGRL3*, *SLC12A2*, *SMIM14*, *SPDEF*, *SPINK1*, *SPINK4*, *ST6GALNAC1*, *TFF3*, *TPM1*, *TPSG1*, *TSPAN13*, *WFDC2*, and *ZG16*. The gene‐set was used to calculate a Goblet cell score (∑(z‐scores)/gene‐set size) for each sample.

### Induction of *Idh1* mutation in mouse intestines

Animal work was carried out in accordance with the Animals in Scientific Procedures Act 1986 (ASPA) under licence *PP3266462*. The conditional knock‐in *Idh1* ^*R132H*^ mutant mouse induces a stem cell and methylator phenotype in the mouse brain [44]. We assessed the effects of mutant *Idh1* in normal mouse intestines by crossing with *Vil1‐Cre*. Mouse genotyping was performed using polymerase chain reaction (PCR) analysis (supplementary material, Table S1). Expression of the mutant allele was confirmed by sequencing mRNA extracted from the mouse intestines, and amplification using PCR primers (FW‐5’‐ATCTTTTGGTGTGTAGGT‐3’, RV‐5’‐GTGACAGGCTGGGTAAAAC‐3’) that produce an ~120 bp product. Assessment of D2HG was not possible in these animals, probably owing to spontaneous degradation of metabolites over time [45]. Methylation levels were assessed in normal intestinal mucosa of knock‐in (*Vil1‐Cre;Idh1* ^*fl(R132H)/+*^, *n* = 12) and control mice (*Vil1‐cre;Idh1* ^*+/+*^, *n* = 5) using Illumina Infinium mouse methylation arrays (Illumina), with the resulting data processed using SeSAMe (https://zwdzwd.github.io/InfiniumAnnotation#mouse, https://bioconductor.org/packages/devel/bioc/vignettes/sesame/inst/doc/nonhuman.html – [46]), leaving 265,710 probes for downstream analysis. Mean CpG methylation *β* values per mouse, which were normally distributed according to the Shapiro–Wilk test (*p* = 0.84), were compared between *Idh1‐*mutant and control animals using linear mixed‐effect model analysis, correcting for age, sex, and BeadChip.

### CRISPR knock‐in of *IDH1* mutation into Caco‐2 cells

The IDH‐wildtype Caco‐2 CRC cell line was cultured in EMEM containing nonessential amino acids, supplemented with 20% FCS, 2 mM L‐glutamine, and 1% penicillin/streptomycin. Knock‐in of *IDH1* ^*R132C*^ (CGT > TGT) and *IDH1* ^*R132G*^ (CGT > GGT) was performed using the CRISPR‐Cas9 HF‐PX459 (V2) plasmid, a gift from Mike McGrew (Addgene, Watertown, MA, USA, #118632) [47]. Intron 4 of *IDH1* was targeted using the spacer sequence 5’‐GTATCTACACCCATTAAGCA(AGG)‐3’. Single nucleotide substitutions were introduced using a homology‐directed repair (HDR) template, containing the desired edit (or wildtype *IDH1* sequence in controls) and the mutated PAM sequence, separated by a floxed neomycin resistance cassette. The HDR template was cloned into an adeno‐associated virus (AAV) serotype 2 expression vector (Cell Biolabs, San Diego, CA, USA). Cells were infected with AAV carrying the HDR template and then transfected with HF‐PX459 using the Neon Transfection System (ThermoFisher Scientific). Positive selection was performed using G418 sulphate. Removal of the neomycin resistance cassette was achieved using Cre Gesicles (Takara Bio, San Jose, CA, USA), followed by isolation of monoclonal populations. Mutant *IDH1* activity was shown by D2HG production using the colorimetric D‐2‐Hydroxyglutarate Assay Kit (Abcam, Cambridge, UK). Methylation levels were assessed using the Illumina 850K BeadChip array (Illumina), using ChAMP for data preprocessing and analysis. For comparison between *IDH1‐*mutant and *IDH1‐*wildtype cells, each biological or technical replicate was treated as a separate data point.

### Statistical analyses

Unless otherwise stated, the co‐occurrence of genetic, molecular, and clinical features in tumours was assessed using Fisher's exact test, *χ* ^2^ test, Student's *t*‐test, or Wilcoxon signed‐rank test. Survival analysis used the R packages ‘*survival*’ (version 3.7–0) and ‘*survminer*’ (version 0.4.9) [48, 49]. A mixed‐effects Cox proportional hazards model (R package ‘*coxme’* (version 2.2–18.1)) [50] was constructed, accounting for age, sex, tumour location and tumour stage, and study as a random effect. Linear mixed‐effect models were constructed using the R packages ‘*lme4’* (version 1.35.1) and ‘*lmerTest’* (version 3.1.3) [51, 52].

## Results

### Genomic features of IDH‐mutant CRCs

We recently formally identified *IDH1* as a CRC driver gene in an analysis of 100kGP cancers [53]. Thirteen CRCs in the 100kGP dataset (version 17) carried mutations at *IDH1* codon R132. Here, taking a hypothesis‐free approach and combining public TCGA‐COADREAD and 100kGP CRC data, we found *IDH2* also to be a CRC driver for the first time using the IntOGen pipeline (https://intogen-plus.readthedocs.io/en/v2024/), principally based on four methods (dNdScv [21], OncodriveFML [22], MutPanning [25], and smRegions [27]) that identified the clustering of missense mutations predicted to be functionally important at codon 172 (z = 2.399, *p =* 0.008; Table 1).

**Table 1:** Clinical features and driver mutations of IDH‐mutant CRCs.

Patient ID	Sex	Age	Histology	Stage	Location	MSI	UC	IDH mutation	APC	TP53	BRAF	KRAS	PIK3CA	POLE
*TCGA‐COADREAD*														
TCGA‐AA‐3555	Female	81	Mucinous	2	Proximal	MSS	*NA*	*IDH1* ^*R132C*^	R499*; R1450*	WT	WT	A146T	WT	P286H
TCGA‐AA‐3556	Male	78	Mucinous	1	Distal	MSS	*NA*	*IDH2* ^*R172S*^	Y935*	R273H	WT	G12R	WT	WT
TCGA‐AA‐3672	Female	90	Adenocarcinoma	3	Proximal	MSI	*NA*	*IDH2* ^*R140W*^	WT	WT	WT	Q61K	WT	WT
TCGA‐F4‐6461	Female	41	Adenocarcinoma	3	Proximal	MSS	*NA*	*IDH1* ^*R132C*^	Q1429*; S943*	WT	WT	G12D	E726K	WT
TCGA‐G4‐6322	Male	65	Mucinous	3	Proximal	MSS	*NA*	*IDH1* ^*R132G*^ (+ *IDH1* ^*I102T*^)	S1356*; Y935*	WT	WT	WT	WT	WT
*S:CORT*														
1	Female	60s	Adenocarcinoma	4	Distal	MSS	*NA*	*IDH1* ^*R132C*^	L1302fs*3	WT	WT	G12V	E453K	WT
2	Male	50s	Adenocarcinoma	4	NA	MSS	*NA*	*IDH1* ^*R132C*^	R564*	WT	WT	G12D	E545K	WT
3	Female	70s	Adenocarcinoma	2	Distal	MSS	*NA*	*IDH1* ^*R132C*^	S1355fs*19; L548fs*1	WT	WT	WT	WT	WT
4	Male	50s	Adenocarcinoma	4	Distal	MSS	*NA*	*IDH1* ^*R132C*^	E578*; E1353*	L194F	WT	WT	R88Q; T1025S	P436S
*Dana‐Farber*														
dfci_2016_228	Female	69	Adenocarcinoma	3	Proximal	MSS	*NA*	*IDH1* ^*R132C*^	R1450*	WT	WT	G12C	WT	WT
dfci_2016_578	Male	61	Adenocarcinoma	2	Proximal	MSS	*NA*	*IDH1* ^*R100Q*^	S1545*; S960*; R2166Q; L2168I; S1864Y; H2149N	R213*	WT	A146T	R88Q; K111N	S459F
dfci_2016_1797	Male	80	Adenocarcinoma	*NA*	Distal	MSS	*NA*	*IDH2* ^*R172S*^	E1286Rfs*3	WT	WT	WT	E542K	WT
dfci_2016_4520	Male	*NA*	Adenocarcinoma	*NA*	Proximal	MSS	*NA*	*IDH2* ^*R172S*^	WT	WT	V600E	WT	WT	WT
dfci_2016_354207	Female	82	Adenocarcinoma	4	Proximal	*NA*	*NA*	*IDH1* ^*R132C*^	P1319Lfs*2	WT	WT	G12V	WT	WT
*MSKCC*														
P‐0007698	Female	57	Adenocarcinoma	4	Proximal	MSS	*NA*	*IDH1* ^*R132C*^	WT	WT	V600E	WT	WT	WT
P‐0013159	Male	74	Adenocarcinoma	1	Rectum	*NA*	*NA*	*IDH1* ^*R132C*^	WT	WT	WT	WT	WT	WT
P‐0013395	Male	73	Adenocarcinoma	4	Proximal	MSS	*NA*	*IDH2* ^*R172K*^	WT	WT	WT	G12V	WT	WT
P‐0013981	Female	80	Adenocarcinoma	3	Proximal	MSS	*NA*	*IDH1* ^*R132C*^	WT	WT	V600E	WT	WT	G1047R
P‐0014116	Female	52	Adenocarcinoma	4	Distal	MSS	*NA*	*IDH2* ^*R172K*^	R232*; I1311Dfs*4	WT	WT	A146T	E542K	WT
*Hartwig*														
1	Male	60s	Adenocarcinoma	*NA*	*NA*	*NA*	*NA*	*IDH1* ^*R132C*^	WT	R150W; R282W	V600E	WT	WT	WT
*QUASAR2*														
1	Female	60s	Adenocarcinoma	2	*NA*	MSS	*NA*	*IDH1* ^*R132C*^	WT	WT	WT	G12D	WT	WT
2	Male	60s	Adenocarcinoma	3	*NA*	MSS	*NA*	*IDH1* ^*R132C*^	*NA*	*NA*	*NA*	*NA*	*NA*	*NA*
*100kGP*														
1	Male	70s	Signet Ring Carcinoma	*NA*	Proximal	MSI	N	*IDH1* ^*R132C*^	WT	WT	V600E	WT	WT	WT
2	Female	70s	Adenocarcinoma	1	Proximal	MSS	N	*IDH1* ^*R132C*^	WT	WT	V600E	WT	WT	WT
3	Female	60s	Adenocarcinoma	3	Distal	MSI	N	*IDH2* ^*R172G*^	L1302fs*	WT	WT	G12V	E545K	WT
4	Female	60s	Adenocarcinoma	2	Proximal	MSI	N	*IDH1* ^*R132C*^	WT	*R273P*	V600E	WT	WT	WT
5	Male	60s	Adenocarcinoma	2	Proximal	MSS	Y	*IDH1* ^*R132C*^	WT	WT	V600E	WT	WT	WT
6	Female	80s	Adenocarcinoma	*NA*	Proximal	MSS	N	*IDH1* ^*R132C*^	R1450*	WT	WT	WT	WT	WT
7	Female	60s	Mucinous	*NA*	Proximal	MSS	N	*IDH1* ^*R132C*^	WT	WT	WT	G12V	WT	WT
8	Male	60s	Adenocarcinoma	3	Proximal	MSS	N	*IDH1* ^*R132C*^	WT	WT	V600E	WT	WT	WT
9	Male	60s	Adenocarcinoma	3	Proximal	MSI	N	*IDH1* ^*R132C*^	WT	K373fs*	V600E	WT	WT	WT
10	Male	60s	Adenocarcinoma	2	Proximal	MSI	N	*IDH1* ^*R132C*^	WT	WT	V600E	WT	WT	WT
11	Male	60s	Mucinous	*NA*	Distal	MSS	N	*IDH1* ^*R132C*^	WT	WT	V600E	WT	WT	WT
12	Male	80s	Adenocarcinoma	NA	Rectum	MSS	N	*IDH2* ^*R172S*^	L409fs*	WT	WT	WT	WT	WT
13	Male	70s	Adenocarcinoma	3	Proximal	MSS	N	*IDH1* ^*R132C*^ (*+* IDH1^D54H^)	WT	WT	V600E	WT	WT	WT
14	Male	60s	Adenocarcinoma	4	Proximal	MSI	N	*IDH1* ^*R132C*^	R216*, R1485fs*	C176R, R273C	WT	WT	WT	WT
15	Male	60s	Adenocarcinoma	*NA*	Distal	MSS	N	*IDH2* ^*R172K*^	E941*, Q1338*	Q165*	WT	WT	WT	WT
16	Male	60s	Adenocarcinoma	3	Distal	MSS	Y	*IDH1* ^*R132C*^	S932fs*, R1450*	WT	WT	G13D	WT	WT

Abbreviations: 100kGP, UK 100,000 Genomes Project; MSI, Microsatellite‐unstable; MSS, Microsatellite‐stable; *NA*, Not available; TCGA, The Cancer Genome Atlas; UC, Ulcerative colitis; WT, wildtype.

We then combined 100kGP and TCGA‐COADREAD with five other CRC genome, exome, or panel sequencing studies: S:CORT, QUASAR2, Hartwig, DFCI and MSKCC. Thirty‐eight CRCs with canonical gain‐of‐function somatic *IDH1* or *IDH2* mutations were found (Table 1), representing a median frequency of 0.58%. There was no significant heterogeneity in the frequency of IDH mutations among cohorts (*p =* 0.87, *χ* ^2^ test; supplementary material, Table S2). Mutations were more common in *IDH1* than *IDH2* (29 versus 9). Amino acid substitutions at other amino acids were almost nonexistent, the only exceptions being noncanonical *IDH1* ^*I102T*^ and *IDH1* ^*D54H*^ mutations co‐occurring with *IDH1* ^*R132G*^ and *IDH1* ^*R132C*^ mutations, respectively, suggesting that the canonical changes found are true drivers [54]. *IDH1* ^*R132C*^ was the most frequent mutation (median 81% of IDH‐mutant CRCs), followed by *IDH2* ^*R172S*^ and *IDH2* ^*R172K*^. All other mutations occurred in single cases (Table 1, supplementary material, Tables S2 and S3).

There was no significant association between IDH mutations and patient age, sex, or stage (*p >* 0.05 for all, data not shown). Two IDH‐mutant patients, both from the 100kGP dataset, had received neoadjuvant chemotherapy prior to analysis and two with available clinical information had inflammatory bowel disease. IDH mutations were not associated with differences in overall survival (supplementary material, Figure S1 and Table S4).

Next, we examined the molecular features of the IDH‐mutant CRCs (Table 1). IDH driver mutations were found to be more prevalent in tumours of the proximal colon (odds ratio [OR] = 2.82; 95% confidence interval [CI] = 1.39–5.71, *p* = 0.004). Twenty‐eight (74%) were microsatellite‐stable (MSS), seven (18%) microsatellite‐unstable (MSI^+^), and the remainder untested or unclassified. There was no association between IDH mutations and MSI (OR = 1.19; 95% CI = 0.52–2.73; *p =* 0.66). *BRAF* ^*V600E*^ mutations were overrepresented in IDH‐mutant cancers (OR = 4.09; 95% CI = 2.07–8.06; *p =* 1.9 × 10^−4^), while *KRAS* driver mutations were not (OR = 1.17; 95% CI = 0.61–2.26; *p =* 0.73). The frequency of pathogenic *APC* mutations was lower in IDH‐mutant CRCs (OR = 0.47; 95% CI = 0.25–0.89; *p* = 0.03). Mutation signature extraction from whole‐genome sequencing data of 16 IDH‐mutant 100kGP CRCs confirmed the presence of the mutation signatures SBS15 and SBS44, associated with MSI, and SBS18, associated with reactive oxygen species DNA damage [55], but no IDH‐specific signature was found (data not shown).

### The methylome of IDH‐mutant CRCs

**Figure 1:**
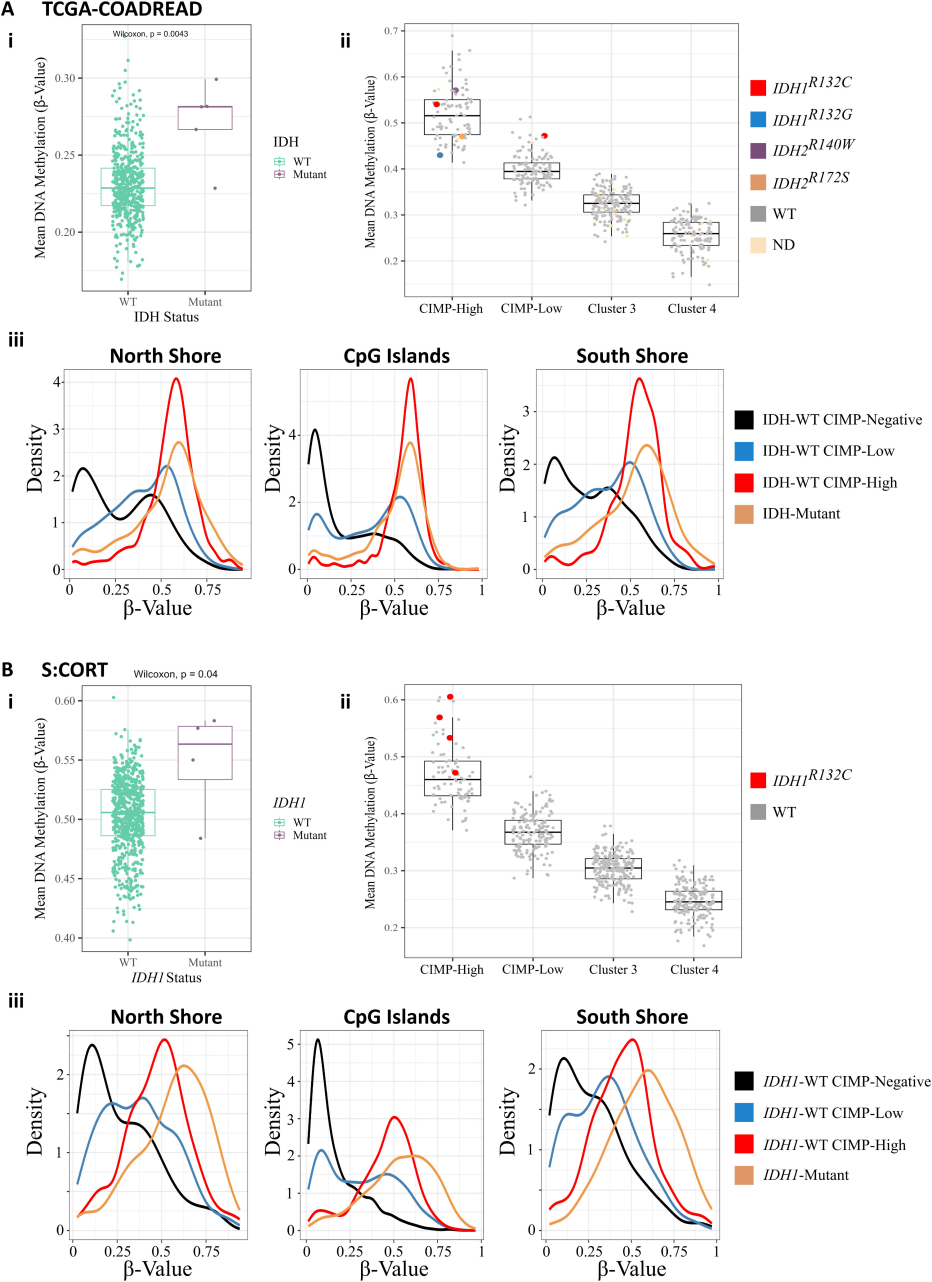
CpG methylation of IDH‐mutant CRCs in (A) TCGA‐COADREAD and (B) S:CORT. (i) The mean DNA methylation probe *β* value per cancer of IDH‐wildtype (WT, green) and IDH‐mutant (purple) CRCs. (ii) Association between global methylation levels (mean probe *β* values) and the four CIMP clusters, calculated from the CpG probes used for RPMM‐based cluster analysis, for each CRC within the four RPMM clusters. CRCs harbouring *IDH1* ^*R132C*^ (red), *IDH1* ^*R132G*^ (light blue), *IDH2* ^*R140W*^ (purple), or *IDH2* ^*R172S*^ (orange) mutations are highlighted, with IDH‐wildtype (WT) cancers in grey. ND = not defined. (iii) DNA methylation *β* value distributions of the probes used for RPMM clustering by IDH mutation status within North Shore (2 kb upstream of CpG island), CpG Island, and South Shore regions (2 kb downstream of CpG island).

Of the 38 IDH‐mutant samples, all 13 with CIMP data were classified as CIMP‐positive (11 CIMP‐high, two CIMP‐low), a strong enrichment for CIMP‐high compared to IDH‐wildtype CRCs (OR = 29.9; 95% CI = 6.6–135.9; *p <* 1 × 10^−4^). We then examined methylation array data from 526 TCGA‐COADREAD CRCs. The mean probe *β* value in IDH‐mutant CRCs (*n* = 5) was significantly greater than that of the 493 IDH‐wildtype cancers (*p =* 0.004; Wilcoxon test; Figure 1A). The same was seen in *IDH1‐*mutant CRCs of the S:CORT study (*n* = 4), which had a significantly greater mean DNA methylation *β* value than *IDH1‐*wildtype CRCs (*n* = 662; *p =* 0.04; Wilcoxon test; Figure 1B). In multivariable regression analysis with study (TCGA‐COADREAD, S:CORT or DFCI) as a random effect, CIMP was independently associated with IDH mutation (OR = 1.63; 95% CI = 1.27–2.09; *p* = 1.3 × 10^−4^), MSI (OR = 1.51; 95% CI = 1.40–1.62; *p* < 2 × 10^−16^), age at diagnosis (OR = 1.004; 95% CI = 1.002–1.006; *p* = 3.2 × 10^−4^) and proximal cancer location (OR = 1.32; 95% CI = 1.26–1.38; *p* < 2 × 10^−16^).

**Figure 2:**
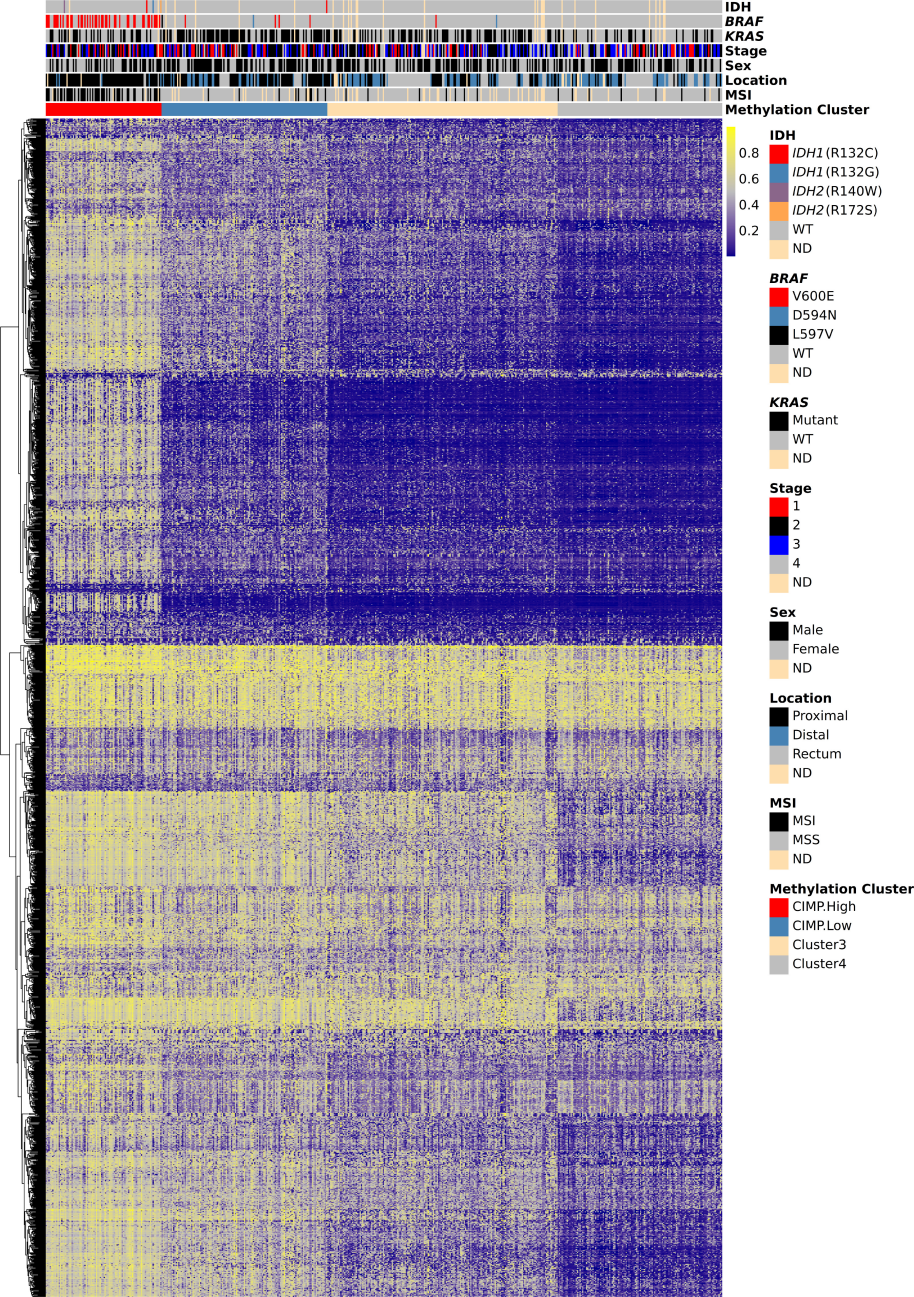
RPMM‐based classification of CIMP status in TCGA‐COADREAD CRCs. *β* values from 2,062 CpG probes (*y*‐axis, where point colour indicates probe *β* value) were subjected to unsupervised recursively partitioned mixture model clustering. The annotation above the heatmap depicts the methylation cluster of each cancer, IDH mutation, and other chosen molecular and clinicopathological features. WT, wildtype; ND, not defined; MSI, microsatellite instability; MSS, microsatellite stable.

**Figure 3:**
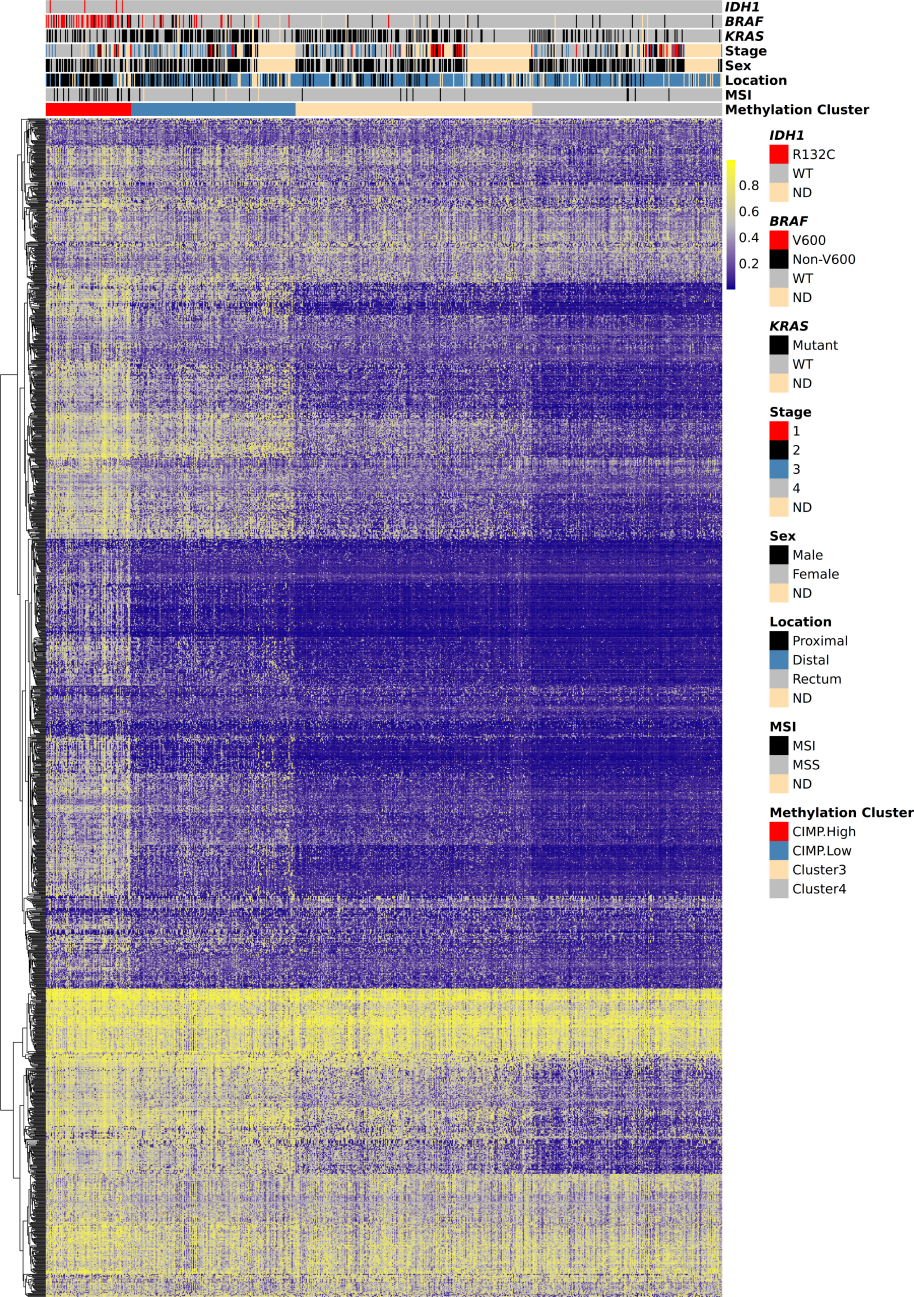
RPMM‐based classification of CIMP status in S:CORT CRCs. Clustering used 1,475 CpG probes. Other features are shown as per Figure 2. WT, wildtype; ND, not defined; MSI, microsatellite instability; MSS, microsatellite stable.

Unsupervised clustering of methylation data from TCGA‐COADREAD (Figures 1A and 2, and supplementary material, Figure S2A) and S:CORT (Figures 1B and 3, and supplementary material, Figure S2B) showed that IDH‐mutant CRCs did not form a discrete group, but were distributed within the larger CIMP‐positive group. The original definitions of CIMP utilised hypermethylation of the promoters of defined panel genes, including *MLH1* and *CDKN2A* [56, 57, 58]. CIMP (type C methylation) was distinguished from age‐related methylation (type A). As larger‐scale analyses of methylation became technically possible, measurement of hypermethylation steadily became based on genome‐wide measures, similar to other tumour molecular features, such as MSI [59]. The term CIMP came to be used synonymously with cluster groups, as we do here, or even with global hypermethylation. We accept that elements of the original ‘CIMP’ may have been lost or altered during this process, but, reassuringly, studies [60] have shown CIMP clusters to be largely concordant with panel‐based CIMP classification [57], despite the use of many non‐CpG island probes in the former.

To determine the effectiveness of genome‐wide methylation clustering compared to CpG island‐specific and CIMP panel‐based approaches, we assessed the mean DNA methylation *β* value of probes mapping to CpG islands in the TCGA‐COADREAD (*n* = 15,665; supplementary material, Figure S3A) and S:CORT (*n* = 125,307; supplementary material, Figure S4A) datasets. The mean DNA methylation *β* value for these probes sequentially increased from Cluster 4 (lowest) to CIMP‐high (highest) in both datasets. Reclustering based on the most variable CpG island probes in the TCGA‐COADREAD dataset (*n* = 1,565) resulted in 515/526 (97.9%) CRCs being assigned to the same methylation cluster as genome‐wide methylation clustering, while reclustering the S:CORT dataset using probes in common with those used in TCGA‐COADREAD clustering (*n* = 1,180) resulted in 642/666 (96.4%) CRCs being assigned to the same cluster as before (data not shown). We then compared the methylation of probes associated with the CIMP panel genes *CACNA1G*, *CDKN2A*, *CRABP1*, *IGF2*, *MLH1*, *NEUROG1*, *RUNX3*, and *SOCS1*, defined by Ogino *et al* [58]. We found that the average DNA methylation of probes associated with these genes again sequentially increased from Cluster 4 (lowest) to CIMP‐high (highest) in TCGA‐COADREAD (supplementary material, Figure S3B–I) and S:CORT (supplementary material, Figure S4B–I) CRCs. Overall, our own exploration shows a clear concordance between panel‐based, CpG island‐specific and genome‐wide methods to classify CIMP in CRC.

### Transcriptome analysis indicates an association between IDH mutation and mucinous histology

**Figure 4:**
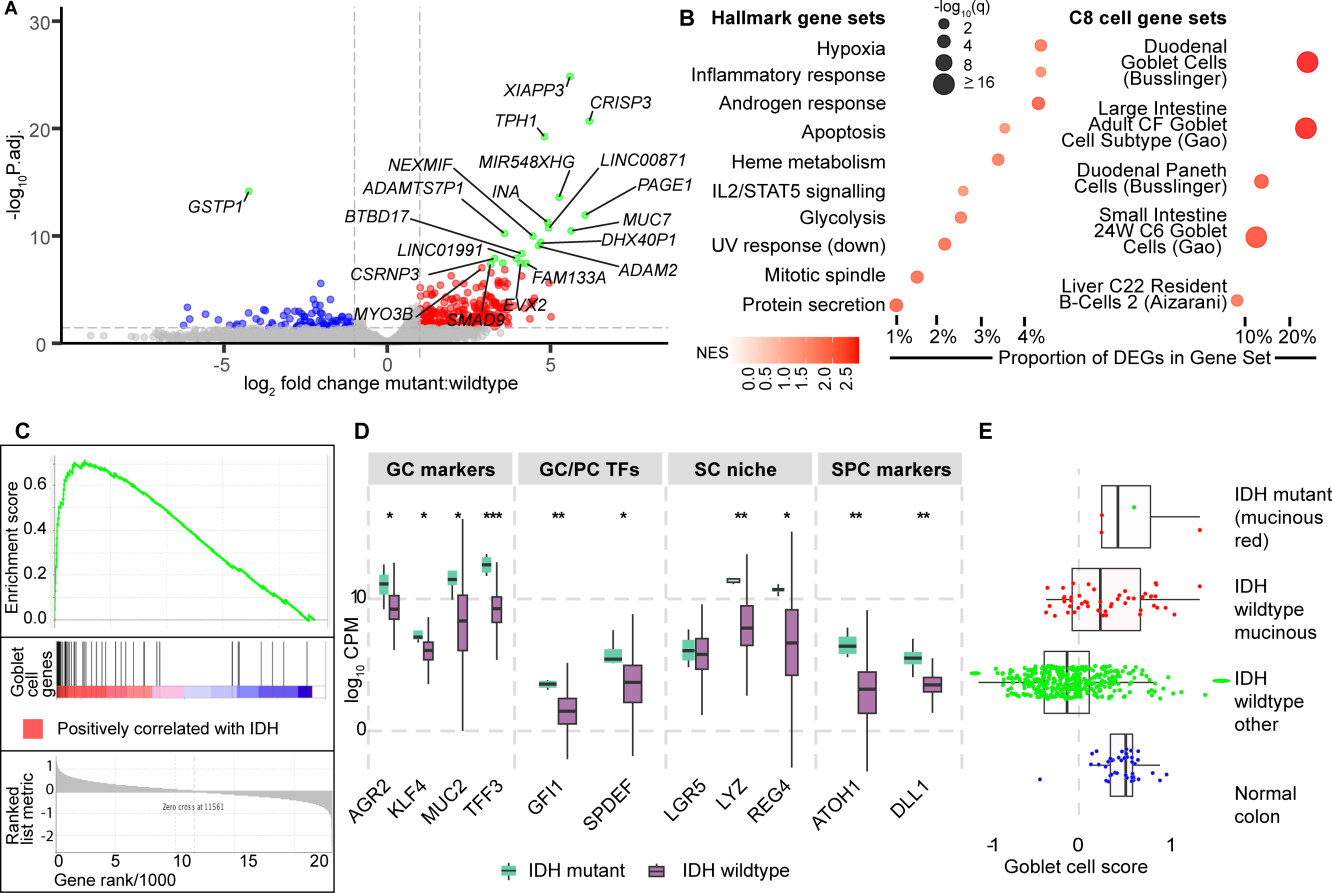
Gene expression characteristics of IDH‐mutant CRCs. (A) Volcano plot of IDH‐mutant (*n* = 4) versus IDH‐wildtype (*n* = 411) CRCs from the TCGA‐COAD study. Differentially expressed genes (DEGs) with *p*_FDR_ *<* 0.05 reflect lower expression (log_2_(fold‐change) < −1, blue) and higher expression (log_2_(fold‐change) > 1, red) in IDH‐mutant versus IDH‐wildtype CRCs, respectively. The top 20 significant DEGs, potential components of an IDH‐mutant gene expression signature, are labelled and coloured green. (B) GSEA normalised enrichment scores (NES) for significant hallmark or cell type gene‐sets from a comparison of IDH‐mutant and IDH‐wildtype CRCs. Proportion of DEGs in gene‐set values indicate the fraction of the gene‐set that is significantly differentially expressed. (C) GSEA for goblet cells showing strong enrichment. (D) Expression levels in IDH‐mutant (purple) and IDH‐wildtype (green) CRCs of selected genes marking secretory progenitors (SPC), Goblet and Paneth cell‐specific transcription factors (GC/PC TFs), Goblet cells (GC markers) and intestinal stem cells (SC niche). Boxes show data between first and third quartiles, with the median as a horizontal line. Whiskers show 1.5× interquartile range. *p < 0.05, **p < 0.01, ***p < 0.001. (E) Goblet cell scores calculated for IDH‐mutant CRCs, colorectal adenocarcinomas with/without mucinous phenotype, and normal colonic tissue samples from TCGA. Tumours reported to have mucinous histology are shown in red, other tumours in green, and normal tissues in blue.

Analysis of TCGA‐COAD CRCs revealed 349 differentially expressed genes (DEGs) (270 up; 79 down) in the four IDH‐mutant CRCs compared to 411 IDH‐wildtypes (Figure 4A). The 20 most significant DEGs were annotated as potential components of a ‘signature’ of mutant IDH in CRC. Genes fulfilling these criteria included *SMAD9*, *EVX2*, and *MUC7* (up) and *GSTP1* (down). GSEA detected positive enrichment of several hallmark gene‐sets in the IDH‐ mutant tumours, including hypoxia, glycolysis, and inflammatory response genes (Figure 4B), and goblet cell genes (Figure 4B,C). We identified differential expression of secretary progenitor (SC) cell‐associated genes, goblet and Paneth cell‐specific transcription factors, Goblet cell markers, and stem cell niche‐associated genes (Figure 4D). Using an *ad hoc* goblet cell score (see Methods), we identified high scores in IDH‐mutant CRCs, comparable to mucinous adenocarcinomas and normal colon (Figure 4E). These findings were consistent with the histological data of Huang *et al* [9]. However, our IDH‐mutant tumours had no excess of CMS3, a CRC subtype associated with mucinous cancers and *KRAS* mutations (OR = 0.25; 95% CI = 0.04–1.49; *p* = 0.15; supplementary material, Table S3) [33]. In multivariable regression analysis with study (TCGA‐COADREAD or S:CORT only, due to a lack of detailed histology data from DFCI) as a random effect, mucinous histology was independently associated with IDH mutation (OR = 1.43; 95% CI = 1.22–1.63; *p* = 7.3 × 10^−4^), MSI (OR = 1.13; 95% CI = 1.06–1.19; *p* = 4.1 × 10^−4^) and proximal location (OR = 1.04; 95% CI = 1.01–1.07; *p* = 0.022).

### Evidence that IDH mutation may causally affect DNA methylation

**Figure 5:**
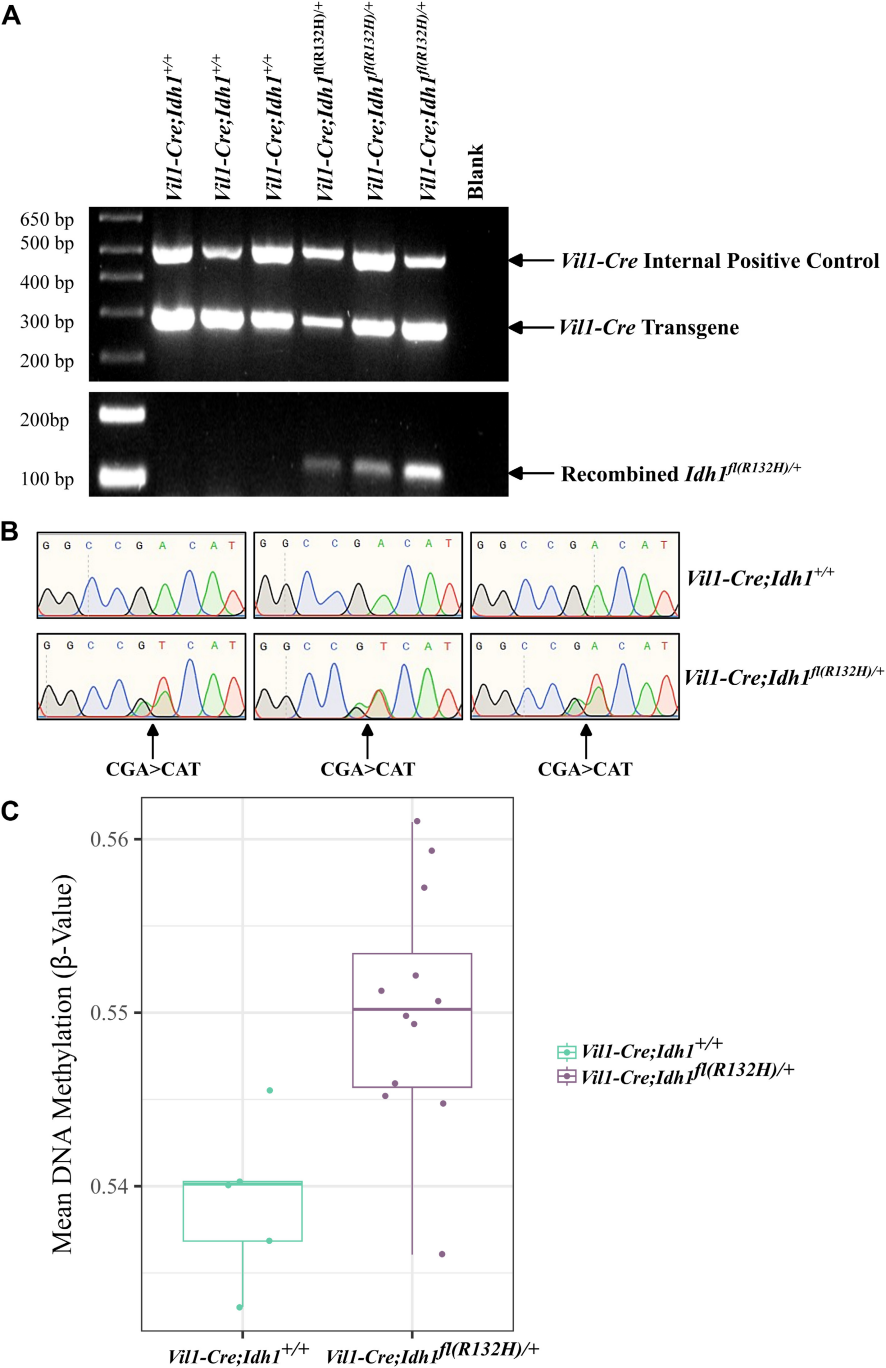
Analysis of *Idh1‐*mutant mice. (A) Genotyping of *Vil1‐Cre* (upper) and the recombined *Idh1* ^*fl(R132H)/+*^ allele (lower) in three *Vil1‐Cre;Idh1* ^*+/+*^ and three *Vil1‐Cre;Idh1* ^*fl(R132H)/+*^ animals. Primer sequences and PCR conditions are provided in the supplementary material, Table S1. (B) *Idh1* RNA sequence derived from intestines of three *Vil1‐Cre;Idh1* ^*+/+*^ mice (upper) and three *Vil1‐Cre;Idh1* ^*fl(R132H)/+*^ mice (lower), showing expression of the mutant allele specifically in the latter. (C) Global methylation (mean *β* value of all probes) in the intestines of test and control *Idh1*‐mutant mice. Individual data points for each mouse are shown as dots. The boxes show the data between the first and third quartiles, with the median as a horizontal line. Whiskers show a maximum of 1.5× interquartile range. A linear mixed effect model, incorporating *Idh1* status, sex, and age as fixed‐effect variables and BeadChip as a random effect was constructed, where mean DNA methylation *β* value (*n* = 265,710 probes) was the outcome variable. Test animals (*n* = 12; purple) were *Vil1‐Cre;Idh1* ^*fl(R132H)/+*^ and control animals were *Vil1‐Cre;Idh1* ^*+/+*^ (*n* = 5; green). The magnitude of the difference between Idh1‐mutant and wildtype mice is small in absolute terms, but its value is likely to be reduced by several factors, including a preponderance of methylation‐invariate sites, a polyclonal cell population and the relatively narrow time window for the *Idh1* mutation to act compared with the life history of a human cancer.

To explore whether IDH mutations were likely to be causal for DNA hypermethylation, we crossed conditional knock‐in *Idh1* ^*fl(R132H)/+*^ mice with animals expressing intestinal Cre (*Vil1‐Cre*). The resulting *Vil1‐Cre*;*Idh1* ^*fl(R132H)/+*^ heterozygotes (Figure 5A,B) displayed no grossly abnormal intestinal phenotype. However, in a linear mixed‐effects model, taking into account *Idh1* status, age, and sex as fixed‐effect variables and BeadChip as a random effect, DNA methylation was modestly, but significantly, increased in the intestinal mucosa of *Vil1‐Cre;Idh1* ^*fl(R132H)/+*^ animals (β = 0.01081, 95 % CI = 0.0042–0.0174; *p* = 0.0043; *n* = 17; Figure 5C). We identified 21,431 DMPs (*p* < 0.05, 94.8% hypermethylated in *Vil1‐cre;Idh1* ^*fl(R132H)/+*^), although none of these remained following multiple testing correction (*p*_FDR_ > 0.05). Whilst direct comparisons are not possible, the order of magnitude of the methylation change (−2%) appeared similar to that found previously when we expressed mutant *Idh1* in the brain in a model of IDH‐mutant glioma [44].

**Figure 6:**
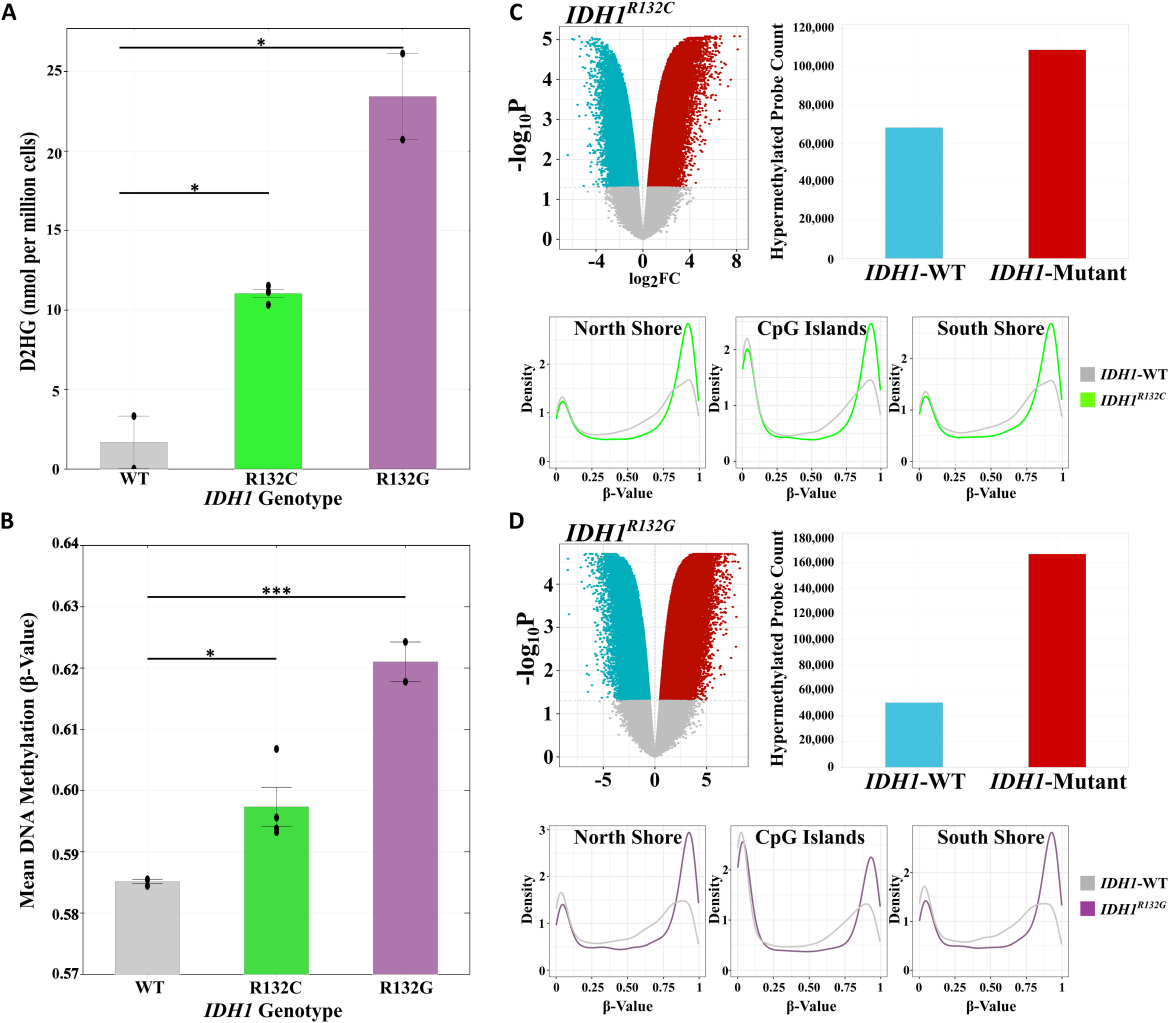
DNA methylation changes in CRISPR‐edited *IDH1*‐mutant Caco‐2 cells. (A) D‐2HG levels for two *IDH1*‐wildtype (WT, grey) replicates, four *IDH1* ^*R132C*^ replicates (comprised of two technical replicates of two independent clones, green), and two replicates of a single *IDH1* ^*R132G*^ clone (purple). *p < 0.05 from two‐tailed Student's *t*‐test for pairwise difference. (B) The mean DNA methylation *β* values of the 728,496 EPIC array probes in Caco‐2 cells, comprising *IDH1*‐wildtype (WT, grey, *n* = 3), *IDH1* ^*R132C*^ (green, *n* = 4), or *IDH1* ^*R132G*^ (purple, *n* = 2). *p < 0.05, ***p < 0.001 from two‐tailed Student's *t*‐test for pairwise difference. Every mutant clone (*n* = 6) had higher methylation than any of the three wildtype clones (*p =* 0.020, Student's *t*‐test). (C, D) Volcano plots and bar charts showing differentially methylated probes in *IDH1* ^*R132C*^ (C) and *IDH1* ^*R132G*^ (D). Plotted in the volcano plots are the log_2_ fold‐change (log_2_FC) in methylation and −log_10_ Benjamini−Hochberg false discovery rate‐corrected *p* value (−log_10_ *P*) for each probe. Hypermethylated probes in *IDH1*‐wildtype cells are shown in turquoise and hypermethylated probes in *IDH1‐*mutant cells in red. Probes in grey show no significant difference between groups (*p*_FDR_ > 0.05). Also shown are the distribution of the *β* values of these significantly differentiated probes located within North Shore (2 kb upstream of CpG island), CpG Island, and South Shore regions (2 kb downstream of CpG island) in *IDH1*‐wildtype (WT, grey), *IDH1* ^*R132C*^ (green), and *IDH1* ^*R132G*^ (purple) Caco‐2 cells.

Previously, Gerecke *et al* [61] found that *IDH1* ^*R132C*^ mutations had no effect on DNA methylation when introduced into the CRC cell line HCT116. However, HCT116 cells are already CIMP‐positive and we reasoned that DNA methylation changes would be more apparent if mutant IDH were expressed in CIMP‐negative cells. We therefore generated CRISPR‐Cas9 knock‐ins of *IDH1* ^*R132C*^ or *IDH1* ^*R132G*^ into CRC cell line Caco‐2 (supplementary material, Figure S5). We confirmed D2HG overproduction (Figure 6A) and analysed DNA methylation. The mean probe *β* value was significantly higher in both the *IDH1* ^*R132C*^ and *IDH1* ^*R132G*^ cells compared to controls (*p =* 0.023 and *p* = 7.0 × 10^−4^, respectively; *t* test; Figure 6B). We identified 177,019 differentially methylated probes in *IDH1* ^*R132C*^ cells (61.5% hypermethylated compared to *IDH1*‐wildtype, Figure 6C; supplementary material, Table S5) and 217,375 in *IDH1* ^*R132G*^ cells (76.8% hypermethylated compared to *IDH1‐*wildtype, Figure 6D and supplementary material, Table S6). In summary, the murine and cell data both supported a direct effect of mutant *IDH1* expression on DNA methylation in intestinal epithelial cells and CRC cell lines.

### Comparison between IDH‐mutant and other CIMP‐positive CRCs

We assessed whether IDH‐mutant CRCs differed from their IDH‐wildtype, CIMP‐positive counterparts. IDH‐mutant CRCs showed no significant differences in the prevalence of *BRAF* ^*V600E*^ (OR = 0.98; 95% CI = 0.48–1.97; *p* > 0.99) mutations, pathogenic *KRAS* mutations (OR = 1.18; 95% CI = 0.60–2.33; *p =* 0.73), MSI (OR = 1.54; 95% CI = 0.66–3.61; *p* = 0.43), or overall survival (hazard ratio [HR] = 0.73; 95% CI = 0.31–1.69; *p =* 0.46; supplementary material, Figure S6 and Table S7).

Despite clustering generally with CIMP‐high cancers (Figures 2 and 3), IDH‐mutant CRCs had borderline significantly higher DNA methylation than other CIMP‐positive CRCs (TCGA‐COADREAD *p* = 0.049 and S:CORT *p* = 0.052, Wilcoxon test; supplementary material, Figure S7A,B). We identified 1,721 DMPs (*p*_FDR_ *<* 0.05) when comparing the two CIMP‐positive groups in the TCGA‐COADREAD. 1,201 (69.8%) of these DMPs were hypermethylated and 520 (30.2%) hypomethylated in the IDH‐mutants (OR = 2.31; 95% CI = 2.01–2.66; *p <* 1 × 10^−4^; supplementary material, Figure S7C and Table S8). Based on suggestions that bivalent promoter hypermethylation was a characteristic of CIMP [62], we found that 436 (25.3%) DMPs mapped to these regions, including 327 hypermethylated (27.2%) and 109 hypomethylated (21%), representing a significant enrichment of the former (OR = 1.41; 95% CI = 1.10–1.81; *p =* 0.007). DMP analysis of the IDH‐mutant S:CORT CRCs revealed 69,748 DMPs (*p*_FDR_ < 0.05), 61,465 (88.1%) were hypermethylated and only 8,283 (11.9%) hypomethylated (supplementary material, Figure S7D and Table S9), reflecting a significant enrichment of hypermethylated probes (OR = 7.42; 95% CI = 7.22–7.63; *p <* 1.0 × 10^−4^).

## Discussion

The CpG island methylator phenotype was first described in CRC and remains best characterised in this tumour type. CIMP is associated with older age, MSI, and *BRAF* mutation [33]. The underlying cause(s) of CIMP in CRC, as well as its downstream functional importance, has remained unidentified. Here, we show using in‐house and publicly‐available datasets, *Idh1*‐mutant mouse models, and CRISPR‐edited cell lines that somatic gain‐of‐function IDH mutations are associated, plausibly causally, with DNA hypermethylation and CIMP in CRC. The DNA methylation profiles of IDH‐mutant and IDH‐wildtype CIMP‐positive CRCs are similar, with a tendency for IDH‐mutants to resemble the CIMP‐high subtype more closely. IDH‐mutant CRCs show mucinous features, including the expression of goblet cell‐related genes, which are generally associated with CIMP. While IDH‐mutant CRCs have been noted previously [10], the small sample sizes of prior studies have precluded firm conclusions being drawn.

In the 100kGP, which, if not unbiased, had very few exclusion criteria for recruitment, we found pathogenic *IDH1* or *IDH2* mutations in 0.58% of CRC patients, a little lower than the frequencies reported previously [8, 9]. While *IDH1* has previously been reported as a driver in CRC, we report driver status for *IDH2* mutations in this malignancy for the first time. Due to this rarity, our analysis combined all *IDH1‐* and *IDH2‐*mutant CRCs into a single group, and thus relied on mechanisms shared between the two genes. Further exploration of the effects of individual *IDH1/2* mutations on DNA methylation is arguably warranted, given that the frequencies of specific *IDH1* and *IDH2* driver mutations vary across other cancer types. Our study finds that IDH‐mutant CRCs are enriched for *BRAF* ^*V600E*^ mutations, but not *KRAS* driver mutations, in agreement with Huang *et al* [9]. We find that 18% of IDH‐mutant CRCs are MSI^+^, a frequency close to that expected by chance [63].

Previous studies have reported IDH‐associated DNA hypermethylation in uncommon malignancies where IDH mutations occur frequently, including AML and glioma [5, 64]. Our analysis of human tumours, mouse models, and cell lines revealed that IDH mutations are associated with DNA hypermethylation in CRCs. This plausibly occurs as a result of D2HG‐mediated inhibition of TET proteins. Therefore, a similar analysis of TET‐mutant cancers would be of future interest. While the functional analysis provided in this study provides indications that the relationship between IDH mutation and DNA hypermethylation may be causal, further molecular characterisation of IDH‐mutant cell lines and mouse models is required to establish this link conclusively.

IDH‐mutant CRCs may benefit from mutant IDH‐inhibitors that are being employed or trialled in other cancer types. Inhibitors of mutant IDH have shown benefit as treatments for AML and glioma, with trials ongoing in cholangiocarcinoma [65, 66, 67]. While DNA demethylating agents have shown limited clinical benefit in the treatment of unselected CRCs [68, 69], inhibition of mutant IDH may not solely act through demethylation. Therefore, despite clear biological differences between CRC and the malignancies with a high prevalence of IDH mutations, cautious exploration of the efficacy of mutant IDH inhibitors and DNA demethylating agents is arguably warranted in IDH‐mutant CRCs.

In conclusion, this study indicates that mutant IDH may drive DNA hypermethylation and CIMP in CRC, and is potentially the first specific cause of CIMP to be identified in this common cancer type. Whilst the aetiology of the majority of CIMP remains unexplained in CRC, the pattern of methylation and gene expression suggest that the mechanism(s) may be shared with IDH, potentially allowing the identification of additional candidate CIMP driver genes in CRC and other cancer types. Whether IDH mutations drive carcinogenesis solely by DNA methylation is unclear, but it seems likely that CIMP can be more than an aging‐associated epiphenomenon.

## Author contributions statement

JCW, MM and JW collated and analysed the genomic data, with support from AANdM, JF‐T, KS, QH, IS, and ST. AF, CB, IL and JM performed analysis on model systems, which were histologically assessed by RKH and MJA. MM, JW and CSH performed the *in vitro* cell line work and associated D2HG assay. CW performed the survival analysis, driver analysis, and RNA‐sequencing analysis (with JW). DK, RK, ED and TM provided samples and data from QUASAR2 and S:CORT. JCW, MM, JW and IT wrote the article, with the approval of all authors. CB initiated and performed the study of mutant *Idh1* in murine intestines. IT designed and supervised the study.

## Supporting information


**Figure S1.** Overall survival of IDH‐wildtype versus IDH‐mutant CRCs
**Figure S2.** Characteristics of the CpG probes used in RPMM clustering
**Figure S3.** Pan‐CpG island and CIMP panel gene DNA methylation of TCGA‐COADREAD CRCs
**Figure S4.** Pan‐CpG island and CIMP panel gene DNA methylation of S:CORT CRCs
**Figure S5.** CRISPR‐Cas9 knock‐in strategy to generate *IDH1* ^*R132C*^ and *IDH1* ^*R132G*^ Caco‐2 cells
**Figure S6.** Overall survival of IDH‐wildtype CIMP‐positive versus IDH‐mutant CRCs
**Figure S7.** Comparing the DNA methylation profiles of IDH‐mutant and IDH‐wildtype CIMP‐positive CRCs in a CIMP‐only analysis

**Table S1.** PCR primers and conditions
**Table S2.** Frequency of canonical *IDH1/2* mutations in CRC datasets
**Table S3.** Clinical features and CIMP statuses of CRCs from public datasets
**Table S4.** Association between overall survival and molecular and clinicopathological variables in CRCs
**Table S5.** DMPs in *IDH1* ^*R132C*^ Caco‐2 cells
**Table S6.** DMPs in *IDH1* ^*R132G*^ Caco‐2 cells
**Table S7.** Association between overall survival and molecular and clinicopathological variables in CIMP‐positive CRCs
**Table S8.** DMPs in IDH‐mutant TCGA‐COADREAD CRCs
**Table S9.** DMPs in *IDH1*‐mutant S:CORT CRCs

## Data Availability

The TCGA‐COADREAD, DFCI and MSKCC datasets are publicly‐available via both the Genomic Data Commons (GDC) data portal (https://portal.gdc.cancer.gov) and cBioPortal (https://www.cbioportal.org/). Sequencing data from the whole S:CORT dataset (Ethical Approval #15/EE/0241) are publicly available in EGA (EGAS00001001521). Additional S:CORT data are available to all academic researchers on submission of a data request to the data access committee. For commercial agencies, the data will be made available through Cancer Research Horizons acting on behalf of the funders and consortium members. Research on the de‐dentified patient data from 100kGP used in this publication can be carried out in the Genomics England Research Environment, subject to a collaborative agreement that adheres to patient‐led governance. All interested readers will be able to access the data in the same manner that the authors accessed the data. For more information about accessing the data, interested readers may contact research-network@genomicsengland.co.uk or access the relevant information on the Genomics England website: https://www.genomicsengland.co.uk/research. Sequencing data from the Hartwig Medical Foundation are freely available for academic use. In order to access this controlled dataset, researchers are invited to submit an access request at https://www.hartwigmedicalfoundation.nl/en/data/data-access-request/. Access to the QUASAR2 dataset (Ethical Approval #04/MRE/11/18) can be requested by contacting Rachel Kerr (rachel.kerr@oncology.ox.ac.uk).
