# Supplementary material for: Analysis of IDH1 and IDH2 mutations as causes of the hypermethylator phenotype in colorectal cancer
Source: J Pathol. 2025 Jun 22;267(1):40–55. doi: 10.1002/path.6446 (PMC12337813; doi:10.1002/path.6446)
Supplement: Supplementary file 1 — Figure S1. Overall survival of IDH‐wildtype versus IDH‐mutant CRCs Figure S2. Characteristics of the CpG probes used in RPMM clustering Figure S3. Pan‐CpG island and CIMP panel gene DNA methylation of TCGA‐COADREAD CRCs Figure S4. Pan‐CpG island and CIMP panel gene DNA methylation of S:CORT CRCs Figure S5. CRISPR‐Cas9 knock‐in strategy to generate IDH1 R132C and IDH1 R132G Caco‐2 cells Figure S6. Overall survival of IDH‐wildtype CIMP‐positive versus IDH‐mutant CRCs Figure S7. Comparing the DNA methylation profiles of IDH‐mutant and IDH‐wildtype CIMP‐positive CRCs in a CIMP‐only analysis [file PATH-267-40-s001.docx]

**Analysis of *IDH1* and *IDH2* mutations as causes of the hypermethylator phenotype** **in colorectal cancer**

JC Ward, M Morgan, J Wood *et al. J Pathol* <https://doi.org/10.1002/path.6446>

**Supplementary Figures** **S1–S7**

**Supplementary Tables S1–S9 (provided as separate Excel file)**


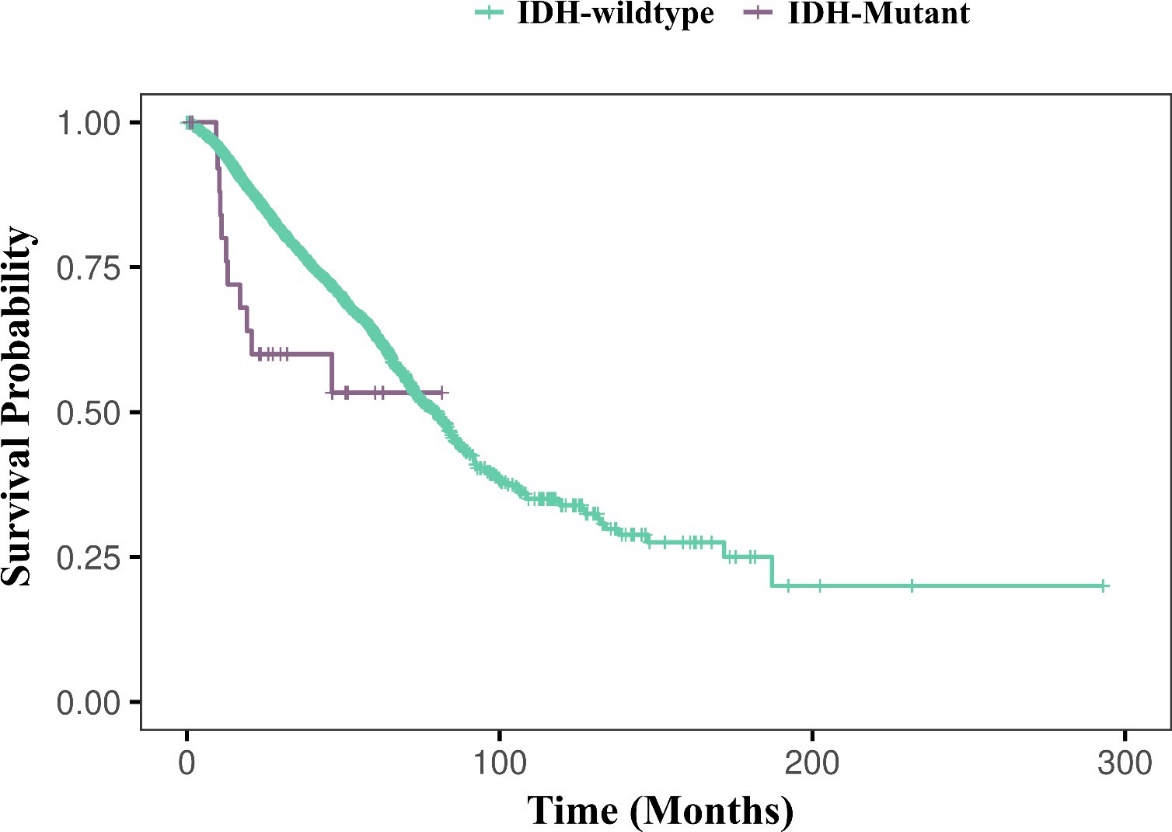


**Figure S1. Overall survival of IDH-wildtype versus IDH-mutant CRCs.** A Kaplan–Meier plot comparing the overall survival (in months) of IDH-wildtype (green) and IDH-mutant (purple) colorectal cancers from the S:CORT [11], QUASAR2 [12], 100kGP [13], TCGA-COADREAD [15] and MSKCC [17] datasets.


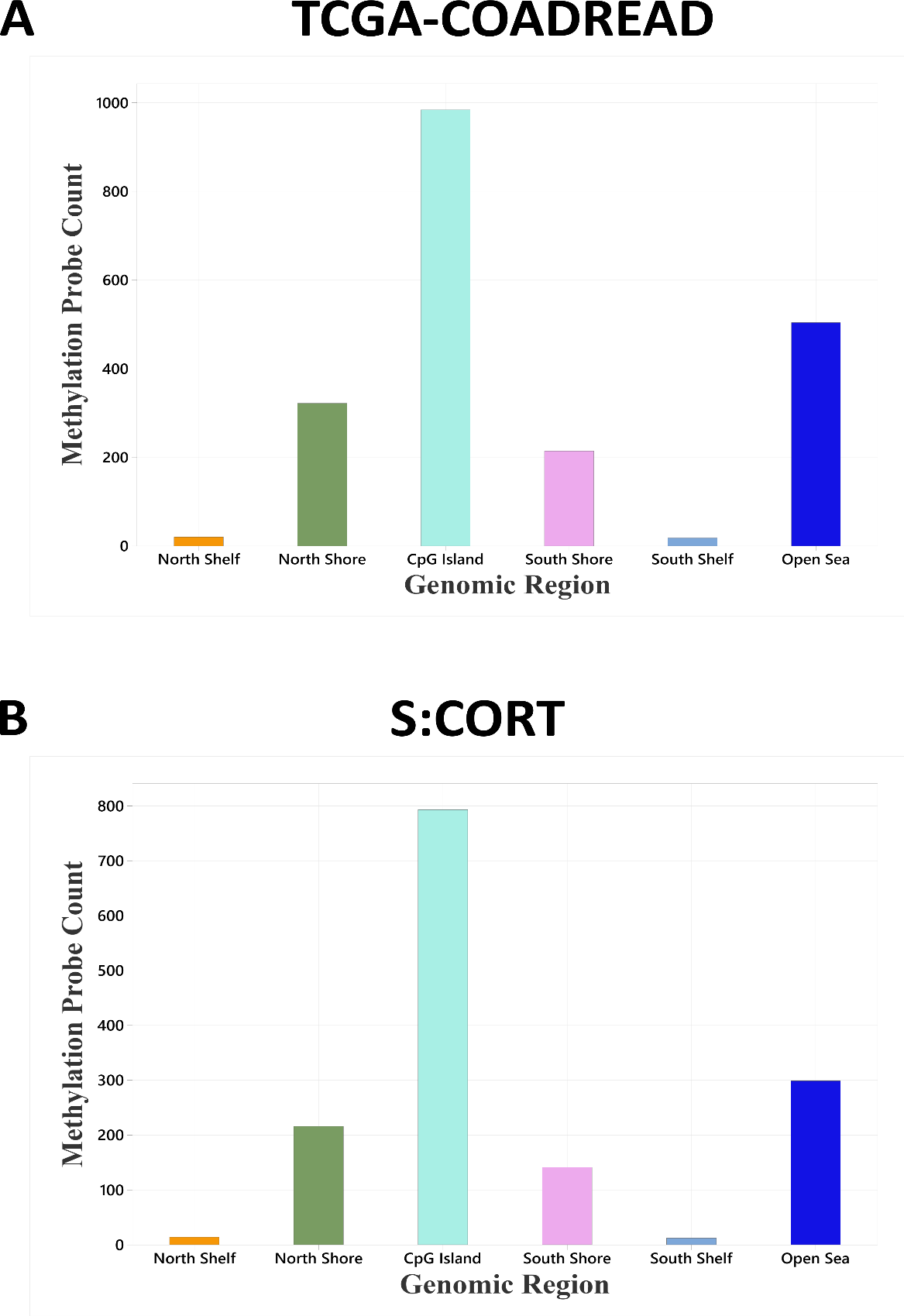


**Figure S2. Characteristics of the CpG probes used in RPMM clustering.** Mapping counts of the CpG probes used in RPMM clustering of the TCGA-COADREAD (A, *n =*2,062) or S:CORT (B, *n =*1,475). Probes were mapped to North Shelf (4 kb upstream of CpG island), North Shore (2 kb upstream of CpG island), CpG islands, South Shore (2 kb downstream of CpG island), South Shelf (4 kb downstream of CpG island) and Open Sea regions.


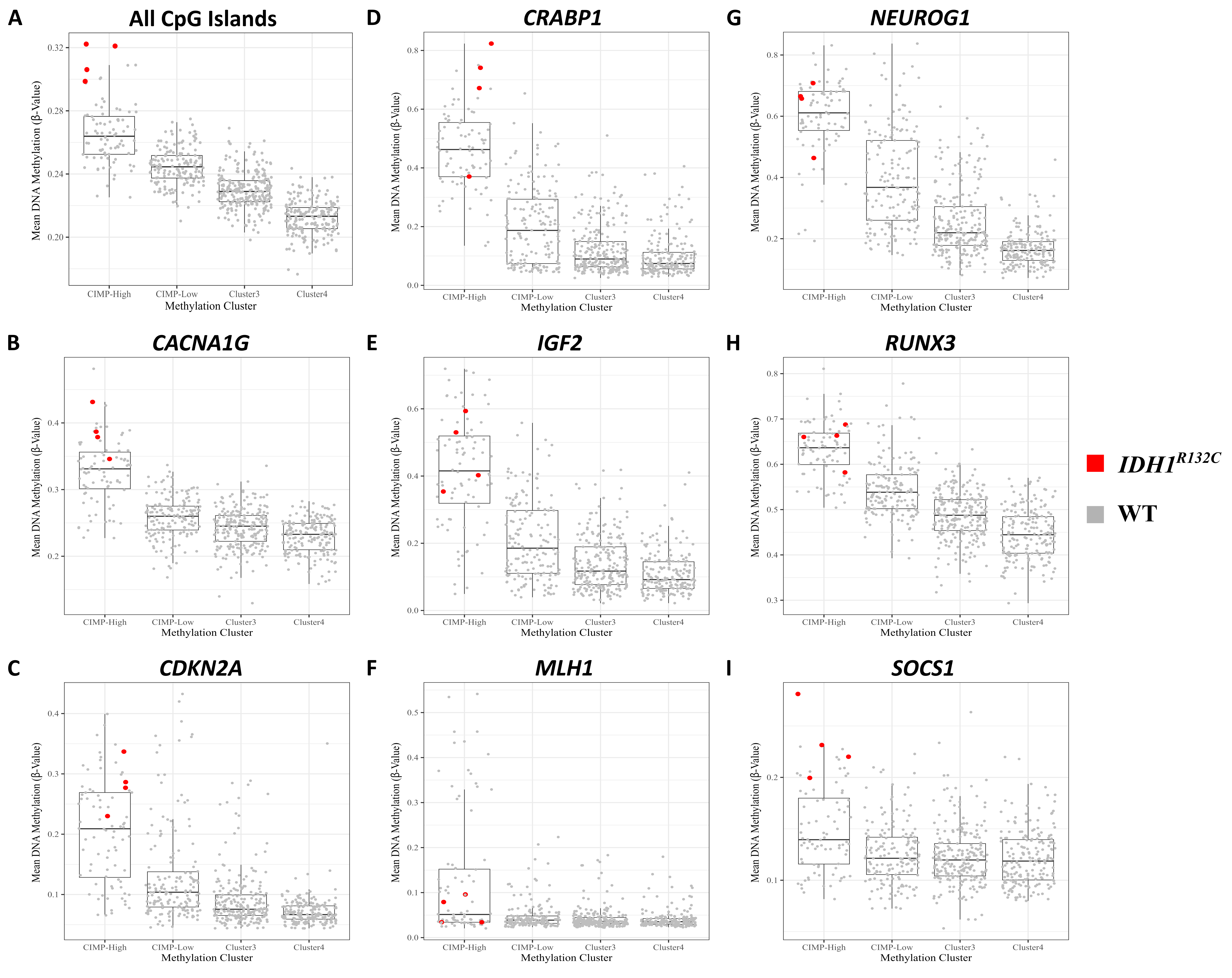


**Figure S3. Pan-CpG island and CIMP panel gene DNA methylation of TCGA-COADREAD CRCs.** (A) The mean DNA methylation β-value of all CpG island-associated methylation probes (*n =*15,665) in TCGACOADREAD CRCs. (B–I) The mean DNA methylation β-value of probes associated with the CIMP panel genes (B) *CACNA1G*, (C) *CDKN2A*, (D) *CRA BP1*, (E) *IGF2*, (F) *MLH1*, (G) *NEUROG1*, (H) *RUNX3,* and (I) *SOCS1*. WT, wildtype; ND, not deﬁned.


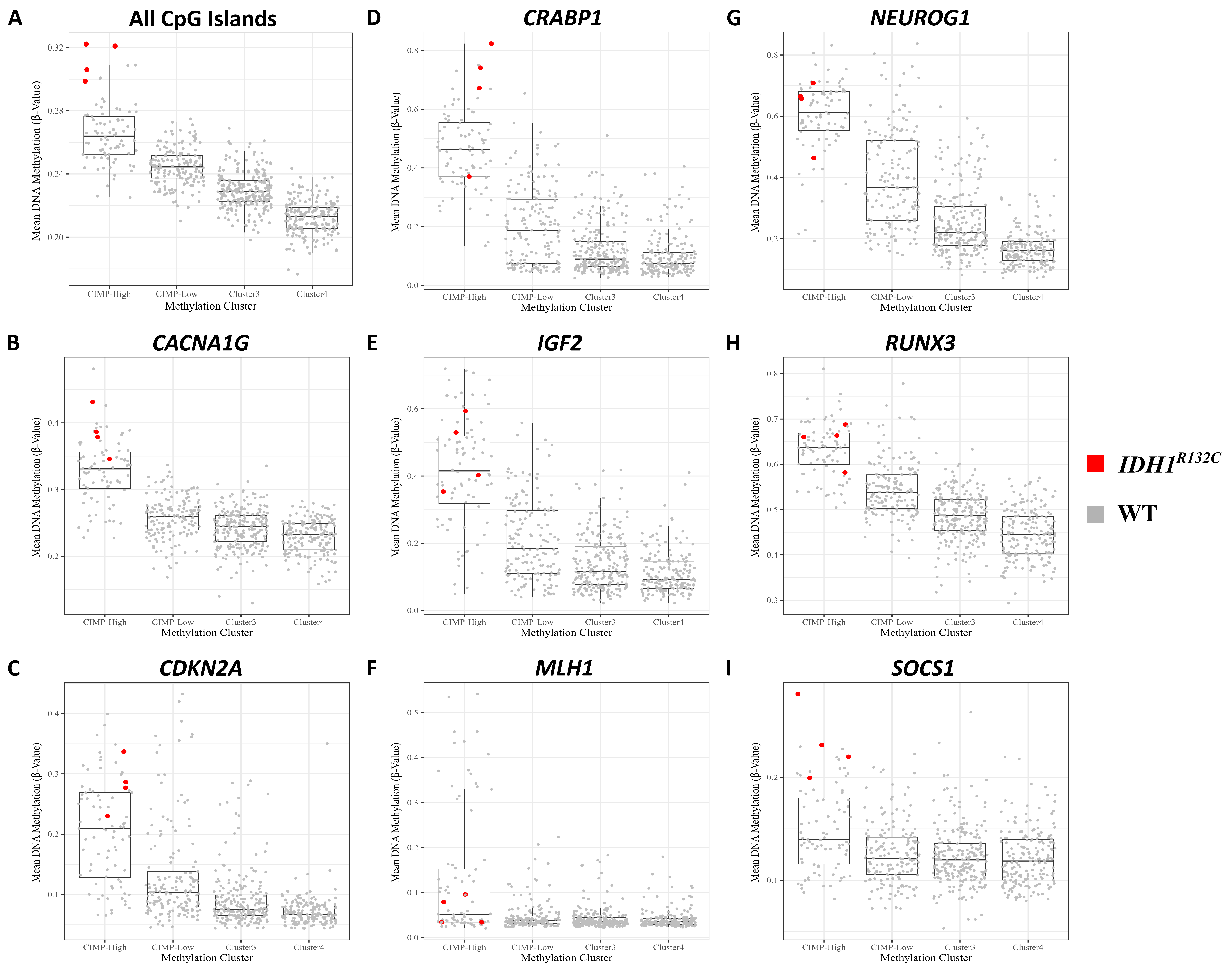


**Figure S4. Pan-CpG island and CIMP panel gene DNA methylation of S:CORT CRCs.** (A) The mean DNA methylation β-value of all CpG island-associated methylation probes (*n =*125,307) in S:CORT CRCs. (B–I) The mean DNA methylation β-value of probes associated with the CIMP panel genes (B) *CACNA1G*, (C) *CDKN2A*, (D) *CRABP1*, (E) *IGF2*, (F) *MLH1*, (G) *NEUROG1*, (H) *RUNX3,* and (I) *SOCS1*. CRCs with *IDH1^R132C^* mutations are indicated in red. WT, wildtype.


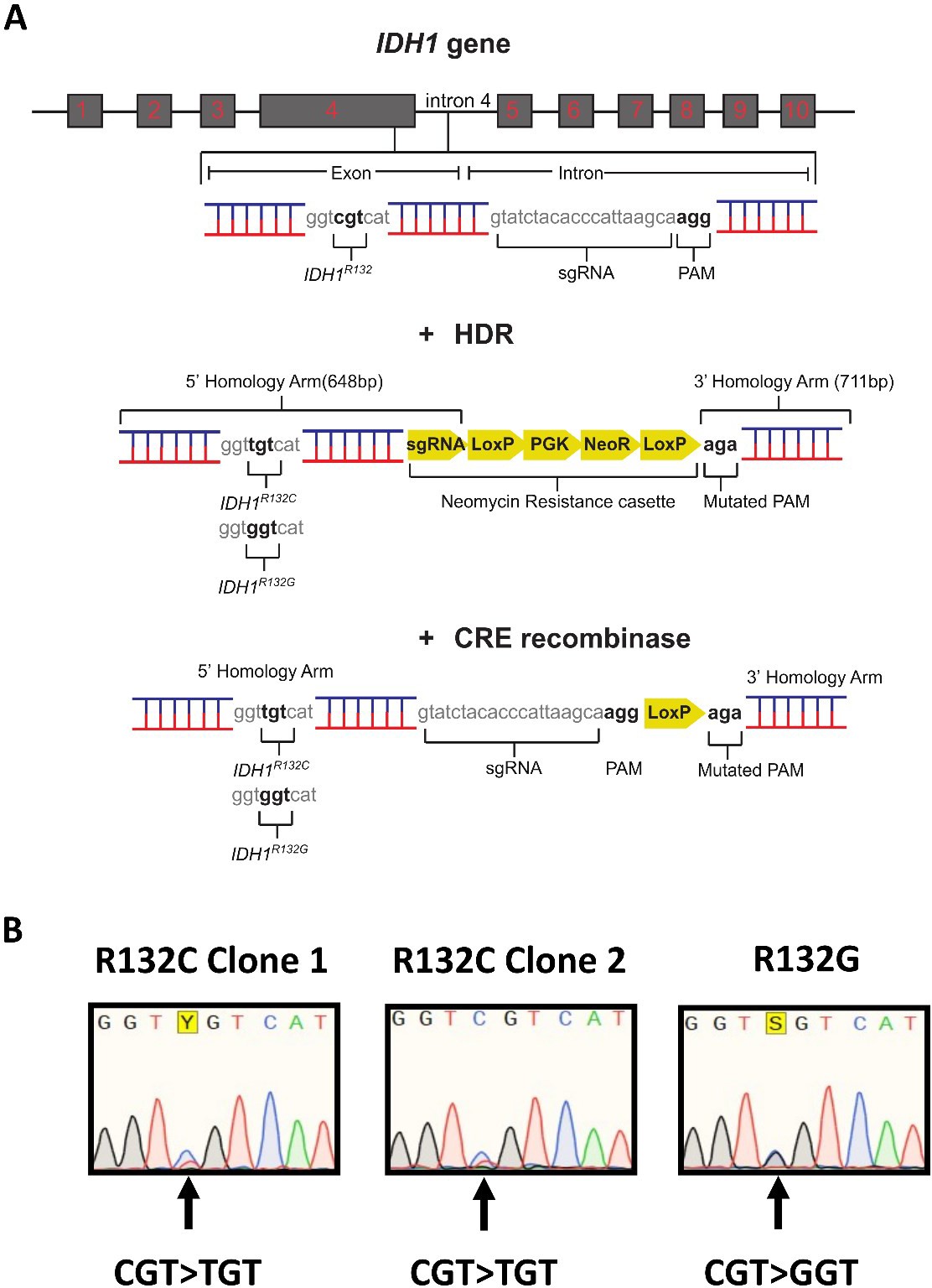


**Figure S5. CRISPR-Cas9 knock-in strategy to generate *IDH1^R132C^* and *IDH1^R132G^* Caco-2 cells.** (A) Schematic representation of the CRISPR knock-in strategy outlining the sgRNA target sequence adjacent to an AGG PAM within *IDH1* intron 4. A homology-directed repair template, consisting of two homologous arms of 648 bp and 711 bp length, a ﬂoxed neomycin resistance cassette, and the nucleotide mutation resulting in *IDH1^R132^*^C^ (CGT>TGT) or *IDH1^R132G^* (CGT>GGT), is delivered via adeno-associated virus. Cells are then positively selected using neomycin prior to removal of the resistance cassette using exogenous *Cre* recombinase. (B) Sanger sequencing traces demonstrating successful introduction of the CGT>TGT change in two Caco-2 *IDH1^R132C^* clones and the CGT>GGT change in a single *IDH1^R132G^* clone.


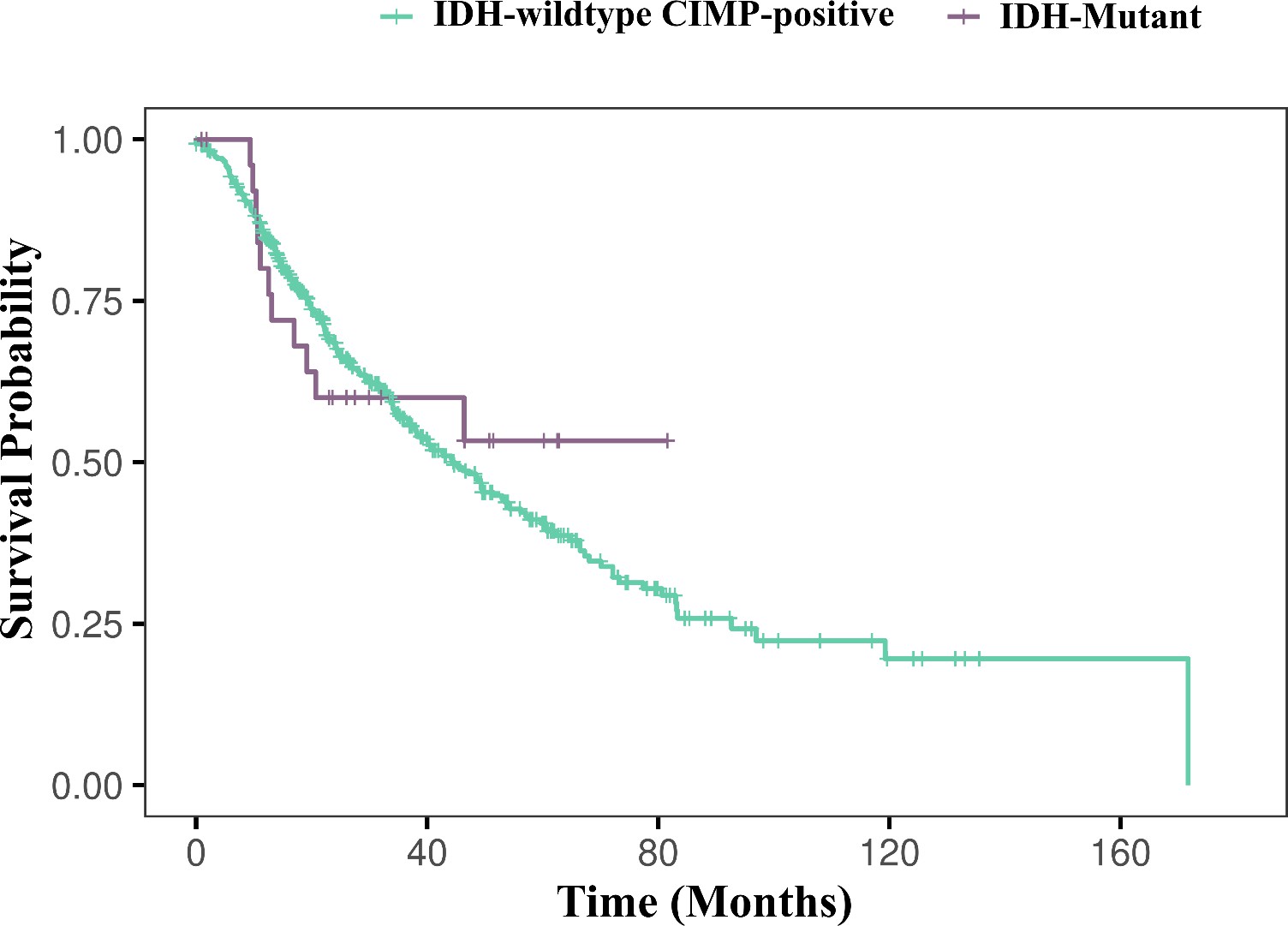


**Figure S6. Overall survival of IDH-wildtype CIMP-positive versus IDH-mutant CRCs.** Kaplan–Meier plot comparing the overall survival (in months) of IDH-wildtype CIMP-positive (green) and IDH-mutant (purple) colorectal cancers from the S:CORT [11], QUASAR2 [12], 100kGP [13], TCGA-COADREAD [15], and MSKCC [17] datasets.


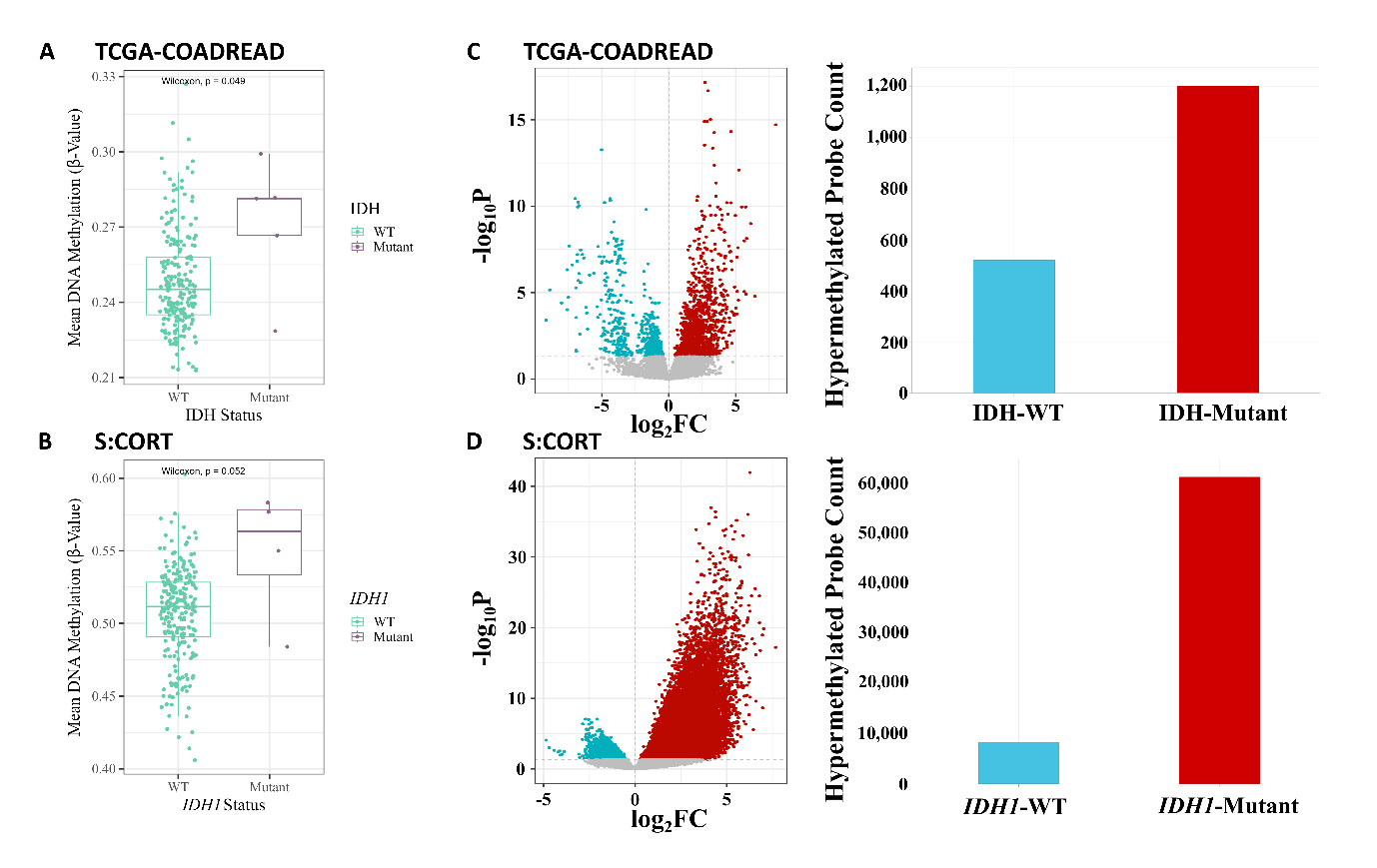


**Figure S7. Comparisons between IDH-mutant and IDH-wildtype CRCs in a CIMP-only analysis.** (A) Distributions of the mean DNA methylation β values per cancer in IDH-wildtype (WT, green) and IDH-mutant (purple) CIMP-positive CRCs from TCGA-COADREAD. (B) A volcano plot and bar chart showing significantly differentially methylated probes in IDH-mutant CRCs compared to IDH-wildtype CIMP-positive CRCs of the TCGA-COADREAD dataset. Plotted for each probe is the log_2_ fold-change (log_2_FC), and the −log_10_ Benjamini-Hochberg corrected *p*-value (−log_10_P). Hypermethylated probes in IDH-wildtype (WT) cancers are shown in turquoise and hypermethylated probes in IDH-mutant cancers in red. Probes in grey show no significant difference between groups (*P_FDR_* > 0.05). (C,D) As for (A) and (B), but data are from S:CORT. (C) The mean DNA methylation β-value per-cancer of *IDH1*-wildtype (WT, green) and *IDH1*-mutant (purple) CIMP-positive CRCs from the S:CORT dataset. (D) A volcano plot and bar chart showing significantly differentially methylated probes in *IDH1*-mutant CRCs compared to *IDH1*-wildtype CIMP-positive CRCs of the S:CORT dataset. Plotted for each probe is the log_2_ fold-change (log_2_FC) and the −log_10_ Benjamini–Hochberg corrected *p*-value (−log_10_P). Hypermethylated probes in *IDH1-*wildtype (WT) cancers are shown in turquoise and hypermethylated probes in *IDH1-*mutant cancers in red. Probes in grey show no significant difference between groups (*p_FDR_* > 0.05).
